# RP11-296E3.2 acts as an important molecular chaperone for YBX1 and promotes colorectal cancer proliferation and metastasis by activating STAT3

**DOI:** 10.1186/s12967-023-04267-4

**Published:** 2023-06-27

**Authors:** Qian Shi, Ying He, Shouyu He, Jingjing Li, Ji Xia, Tianwei Chen, Lixia Huo, Yuhang Ling, Qinchen Liu, Wei Zang, Qiang Wang, Chengwu Tang, Xiang Wang

**Affiliations:** 1 Key Laboratory for Translational Medicine, First Affiliated Hospital, The First People’s Hospital of Huzhou, Huzhou University, Huzhou, 313000 China; 2 Key Laboratory of Integrated Oncology and Intelligent Medicine of Zhejiang Province, Affiliated Hangzhou First People’s Hospital, Zhejiang University School of Medicine, Hangzhou, 310006 China; 3 Medical Transformation Research Institute, The First Affiliated Hospital of Anhui Medical University, Hefei, 230000 China; 4 Department of Hepatobiliary Surgery, The First Affiliated Hospital of Anhui Medical University, Hefei, 230000 China; 5 Key Laboratory of Nutrition, Metabolism and Food Safety, Shanghai Institutes for Biological Sciences, Chinese Academy of Sciences, 320 Yueyang Road, Shanghai, 200031 China; 6 Department of General Surgery, Shanghai Fengxian Central Hospital (Affiliated Fengxian Hospital to Southern Medical University), Shanghai, 201499 China

**Keywords:** CRC, Metastasis, Proliferation, RP11-296E3.2/YBX1, STAT3 transcription

## Abstract

**Background:**

RP11-296E3.2 is a novel long noncoding RNA (lncRNA) associated with colorectal cancer (CRC) metastasis, that was reported in our previous clinical studies. However, the mechanisms of RP11-296E3.2 in colorectal tumorigenesis remain elusive.

**Methods:**

RNA sequencing (RNA-seq), Fluorescence in situ hybridization (FISH), Transwell assays and others, were performed to evaluate the function of RP11-296E3.2 for proliferation and metastasis in vitro. In situ and metastatic tumor models were performed to evaluate the function of RP11-296E3.2 for proliferation and metastasis in vivo. RNA-pulldown, RNA-interacting protein immunoprecipitation (RIP), tissue microarray (TMA) assay, a luciferase reporter assay, chromatin immunoprecipitation (ChIP) and others were performed to explore the mechanisms by which RP11-296E3.2 regulates CRC tumorigenesis.

**Results:**

RP11-296E3.2 was confirmed to be associated with CRC cell proliferation and metastasis in vitro and in vivo. Mechanistically, RP11-296E3.2 directly bound to recombinant Y-Box Binding Protein 1 (YBX1) and enhanced signal transducer and activator of transcription 3 (STAT3) transcription and phosphorylation. YBX1 promoted the CRC cell proliferation and migration, while knockdown of RP11-296E3.2 attenuated the effects of YBX1 on CRC cell proliferation, and metastasis and the expression of several related downstream genes. We are the first to discover and confirm the existence of the YBX1/STAT3 pathway, a pathway dependent on RP11-296E3.2.

**Conclusion:**

Together, these novel findings show that the RP11-296E3.2/YBX1 pathway promotes colorectal tumorigenesis and progression by activating STAT3 transcription and phosphorylation, and suggest that RP11-296E3.2 is a potential diagnostic biomarker and therapeutic target in CRC.

**Supplementary Information:**

The online version contains supplementary material available at 10.1186/s12967-023-04267-4.

## Background

CRC is the third most common type of cancer diagnosed worldwide. It is the fifth leading cause of cancer deaths in China and the second leading cause of cancer-related deaths globally. Since the clinical symptoms of CRC are atypical, approximately 15% of patients have already developed liver metastases at the time of diagnosis. Metastatic colorectal cancer (mCRC) is a thorny clinical problem. Approximately 35–45% of mCRC patients are subject to recurrence within 5 years after surgery. Although continuous advances in surgical techniques and treatments have improved survival outcomes, patients with metastasis and recurrence still have a poor prognosis [1]. Therefore, there is an urgent need to identify the underlying mechanisms of mCRC and develop strategies for early diagnosis and optimal therapy of mCRC.

Numerous studies have shown that protein-coding genes are responsible for cancer metastasis, but the importance of lncRNAs in regulating tumor metastasis is now being realized. LncRNAs are a type of non-coding RNAs that have a length of more than 200 bp and are present in both the cytoplasm and nucleus, which allows them to participate in biological processes [2, 3]. For example, colon cancer-associated transcript 1 (CCAT1) was found to be significantly overexpressed in cancer cell lines and tissues [4], particularly in tumors with lymph node metastasis, and Rg3 was found to reduce the metastatic ability of Caco-2 cells in vitro by suppressing CCAT1 [5]. To identify the metastasis-associated lncRNAs in CRC, we first verified a series of lncRNAs identified in a bioinformatics analysis performed by Shen et al. [6] by examining the tissues and plasma of clinical patients. We found that the expression of a new lncRNA -RP11-296E3.2 was strongly associated with metastasis in CRC patients. Moreover, this research revealed that RP11-296E3.2 had higher sensitivity and specificity for the diagnosis of CRC metastasis than did CEA [7].

In this study, the exact sequence length of RP11-296E3.2 was first determined. RNA-seq analysis revealed that RP11-296E3.2 was associated with CRC cell proliferation and metastasis, and in vitro and in vivo studies further confirmed the oncogenic role of RP11-296E3.2. Furthermore, we found that RP11-296E3.2 could bind to YBX1 directly and function as a crucial molecular chaperone to activate the transcription and phosphorylation of STAT3, resulting in the tumorigenesis and progression of CRC. In summary, our study elucidates a novel mechanism for RP11-296E3.2 in tumorigenesis and suggests that it could be used as a diagnostic biomarker and therapeutic target for CRC.

## Methods

### Cell lines and antibodies

The HCT116, RKO, SW48, HT29, SW620 and 293T cell lines were purchased from the Cell Bank of Type Culture Collection of the Chinese Academy of Sciences, Shanghai Institute of Cell Biology, Chinese Academy of Sciences. HCT116 cells were cultured in McCoy’s 5A medium (Gibco, Carlsbad, CA, USA) supplemented with 10% fetal bovine serum (FBS; Gibco, Carlsbad, CA, USA). The other cell lines were cultured in DMEM (Gibco) supplemented with 10% FBS. All cells were cultured at 37 °C in a humidified incubator with 5% CO_2_.

The antibodies are listed in Additional file 6: Table S3.

### FISH and immunofluorescence assays

The RP11-26E3.2 and U6 (nuclear marker) probes were synthesized by and purchased from RiboBio. According to the manufacturer’s recommendations, FISH was carried out using a Ribo™ Fluorescent in Situ Hybridization Kit (RiboBio, Guangzhou, China). First, 4% paraformaldehyde was used to fix tumor cells (HCT116 and RKO) after they were seeded in a confocal dish. Then, the cells were permeabilized using 0.5% Triton X-100. Nonspecific background staining was blocked by incubating the cells at 37 °C for 30 min before the addition of prehybridization solution. The RP11-26E3.2 and U6 probes were used for hybridization, which was performed overnight. DAPI was used to label the cells after they were washed, and images were acquired using a confocal microscope (Zeiss, Cambridge, UK).

For immunofluorescence staining, cells grown on chamber slides were fixed with 4% formaldehyde/PBS for 10 min at room temperature (RT), and permeabilized with 0.5% Triton X-100/PBS for 10 min, and nonspecific antibody binding was then blocked by incubation with 5% FBS/PBS for 30 min at RT. Then, the cells on the slides were stained with the designated primary antibodies and fluorescent secondary antibodies (#A31572; Invitrogen, Carlsbad, CA, USA), and images were acquired using a confocal microscope.

### Rapid amplification of cDNA ends (RACE)

A HiScript-TS 5′/3′ RACE Kit (#RA101-01; Vazyme, Nangjing, China) was used in accordance with the manufacturer’s instructions to perform 5′-RACE, and 3′-RACE of RP11-296E3.2. A 5 min TA/Blunt-Zero Cloning Kit (#C601; Vazyme) was used to generate transformants and the sequences amplified by 5′-RACE, and 3′-RACE were confirmed by sequencing. Additional file 6: Table S1, a list of the gene-specific primers used for RACE.

### siRNAs, plasmids and stable cell lines

Double-stranded RNA oligonucleotide small interfering RNA (siRNAs) were designed and synthesized by a biological supply company (Biomics, Jiangsu, China) and were ligated into the pLKO.1 plasmid for subsequent short hairpin RNA (shRNA) generation. The target sequences are listed in Additional file 6: Table S2. The P23 plasmid was used to construct the RP11-296E3.2 and YBX1 overexpression plasmids. The target plasmids or GFP (pHAGE-fEF1a-IRES-ZsGreen vector), along with psPAX2 and pMD2.G, were transfected into HEK293T cells for lentivirus packaging using Lipofectamine 3000 (#L3000015; Invitrogen, Carlsbad, CA, USA). Stable cell lines were constructed by incubation with supernatants containing lentiviral particles. Then CRC cell lines were selected by puromycin treatment (pLKO.1 vector; 3 days or longer) or flow sorting.

### RNA-seq and functional pathway analysis

RKO cells were transduced with the sh-Con (n = 3), sh-RP11-296 #1/2 (n = 3) or Vector (n = 3) plasmids or with the RP11-296E3.2-OE (n = 3) plasmid. Total RNA was isolated from these 5 groups of cells with TRIzol reagent (#15596018; Invitrogen, Life technologies, USA). RNA samples subjected to poly(A)RNA sequencing (Poly(A)-seq) analysis according to the manufacturer’s guidelines. In brief, raw data were generated and DESeq, DEGseq and edgeR were used to identify genes with substantial differential expression.

Gene set enrichment analysis (GSEA), Gene Ontology (GO) term analysis and Kyoto Encyclopedia of Genes and Genomes (KEGG) pathway analysis were employed for functional pathway analysis. Significant differentially expressed genes (SDEGs) identified by the criteria |log2FC| > 1 and P_adj_ < 0.05 between the vector and RP11-OE or the sh-Con group and the two sh-RP11 groups. GO and KEGG terms enrichment for significant SDEGs using online DAVID analysis (https://david.ncifcrf.gov/summary.jsp). GSEA was performed with all genes using GSEA4.3.2 software, and complete results of the GSEA is shown in Additional files 7.

### Cell proliferation assay

Two methods were used to evaluate cell proliferation. For real time cellular analysis (RTCA, xCELLigence system, ACEA Biosciences, San Diego, USA), a microelectronic cell sensor chip was integrated into the bottom of a cell detection board to construct a real-time, dynamic and quantitative cell impedance detection sensor system to track changes in cell proliferation and differentiation. A total of 1 × 10^4^ cells were seeded in 16-well plates and cultured for 72 h, and the proliferation curve was then generated by a computer. For the colony formation assay, 3 × 10^3^ transfected HCT116 cells and 5 × 10^3^ transfected RKO cells were plated in each well of 6-well plates. The colonies were then fixed with methanol, prior to staining procedure with 0.1% crystal violet (#548629; Sigma, St Louis, MO, USA) in PBS for 15-min. The number of stained colonies was used to quantify colony formation.

### Cell migration and invasion assays

Transwell was used to measure migration and invasion abilities of CRC cells. In brief, fresh culture medium was added to the bottom chambers. The untreated upper chambers were filled with CRC cells (5 × 10^5^) suspended in serum-free medium for the migration assay. For the invasion assay, the upper chambers were pretreated with Matrigel (BD Bioscience, San Jose, CA, USA) and seeded with the same number of CRC cells The same number of CRC cells. The migrated and invaded cells were observed and counted in at least three random fields after thorough washing, fixation, and crystal violet staining. In addition, a three-dimensional (3D) tumor spheroid invasion assay was performed to evaluated the invasion ability of CRC cells. Cells were counted using a hemocytometer, and diluted cell suspension (1 × 10^4^) was seeded in ultralow attachment (ULA) 96-well plates to generate tumor spheroids of 300–500 µm in diameter. Then, Matrigel was added to the wells of an ULA 96-well plate, and tumor spheroids were placed in the center of the wells. After 1 week, a typical “starburst” invasion pattern was obtained observable using an inverted microscope [8].

### Studies in nude mice

Male nude mice (BALB/c; 6 weeks old) were purchased from the Shanghai SLAC Laboratory Animal Co. Ltd. and used in the mouse model. Over the course of the study, six mice per cage were housed and kept in a pathogen-free barrier environment (approximately 20 °C with 40% humidity and a 12-h day/night cycle). To establish tumor xenograft models, the generated stable HT29 cells (sh-Con, sh-RP11-296 #1 and sh-RP11-296 #2) were injected subcutaneously into nude mice (5 × 10^6^ cells per mouse; n = 5 per mice group). Then, the nude mice were sacrificed 4 weeks after cell inoculation, and the tumors were removed, weighed, and subjected to HE and Ki67 staining. To establish liver metastasis models, the generated stable HT29 cells (sh-Con, sh-RP11-296 #1 and sh-RP11-296 #2) were injected into the hepatic portal vein [9] of nude mice (5 × 10^6^ cells per mouse; n = 4 mice per group). Liver metastases were assessed by bioluminescence imaging (IVIS Lumina XR, Caliper Life Science, MA, USA) each week. After 6 weeks, mice were sacrificed and the livers were removed for PCR analysis and HE staining.

The Animal Care and Use Committee of the First Affiliated Hospital of Huzhou University approved all animal research (approval number: 2021KYLL-Y-008), which was carried out in accordance with the Handbook for the Care and Use of Laboratory Animals. The maximum tumor size was limited by the animal welfare policy to 2 cm. In this investigation, the maximum tumor size was not reached.

### RNA pulldown and mass spectrometry (MS)

In brief, the full-length sense and antisense RP11-296E3.2 sequences, including the T7 promoter, were first synthesized using the TranscriptAid T7 High Yield Transcription Kit (#K0441; Thermo Fisher Scientific, USA). Then, the transcribed RNA (sense and antisense) was biotinylated (#20160; Pierce™ RNA 3′ End Biotinylation Kit, Thermo Fisher Scientific, USA). Pierce Magnetic RNA-Protein Pull-Down Kit (#20164, Thermo Fisher Scientific, USA) was used for the RNA pulldown assay in accordance with the manufacturer's instructions. Western blotting (WB) and MS were used to analyze the isolated proteins.

### RNA immunoprecipitation (RIP) assay

A MagnaRIP RNA-Binding Protein Immunoprecipitation Kit (#17-700; Millipore, Darmstadt, Germany) was used to carry out the RIP assay in accordance with the manufacturer's instructions. In brief, anti-IgG antibody, anti-YBX1 antibody (CST) or anti-ELAVL1antibody (CST) was coupled to magnetic beads, and the beads were incubated with the corresponding cell lysates at 4 °C overnight. Following protein digestion with proteinase K (Sigma, St Louis, MO, USA), RNA was extracted using phenol–chloroform. To measure the expression of RP11-296E3.2, reverse transcription and real-time quantitative polymerase chain reaction (qRT-PCR) were carried out.

### Clinical samples and study approval

A total of 195 paraffin blocks of CRC patient tissue were obtained between 2018 and 2019. The CRC samples were used to construct a CRC tissue microarray (TMA), which was then scanned, photographed, and scored using a Vectra2 analysis system (PerkinElmer, Waltham, MA, USA). Blood samples were obtained from another 59 patients with CRC and 32 healthy participants (healthy volunteers undergoing a physical examination in our facility) between 2019 and 2021 for exosome research.

All experiments were conducted with the approval of the Ethics Committee of the First Affiliated Hospital, Huzhou University (approval number: 2021KYLL-Y-008). All patients signed informed consent forms, and the Ethics Committee gave their approval for to all investigations. Every methodology complied with the requirements outlined in the Declaration of Helsinki. The parameters of the thorough pathological examination were documented and are accessible for analysis.

### Dual luciferase reporter assay

The PGL3-STAT3 promoter plasmid was purchased from Tsingke (Beijing, China). A dual-luciferase reporter kit (#E2920; Promega, WI, USA) was used for the luciferase reporter assay. Following the manufacturer’s instructions. Luciferase activity was determined by normalizing the value of Renilla luciferase luminescence to that of firefly luciferase luminescence by using Dual Luciferase Reporter Assay (#E1910; Promega, Madison, WI, USA). All transfections were performed in triplicate.

### Chromatin immunoprecipitation (ChIP) assay

An EZMagna ChIP A/G Kit was used for the ChIP assay (#1710086; Millipore, MA, USA). A total of 4 × 10^6^ cells were fixed with 1% formaldehyde for 10 min at room temperature (RT), and nuclei were then separated using nuclear lysis buffer supplemented with a protease inhibitor. Chromatin DNA was sheared into fragments with lengths ranging from 100 to 200 bp using a sonicator. The sheared chromatin was immunoprecipitated at 4 °C overnight with an anti-YBX1 antibody. An anti-RNA pol II antibody (Millipore MA, USA) was used as the positive control and regular mouse IgG was utilized as the negative control. The ChIP-qPCR primers are listed in Additional file 6: Table S1. The experiments were repeated independently three times.

### Weighted correlation network analysis (WGCNA)

The transcriptome data were downloaded from The Cancer Genome Atlas-Colorectal Adenocarcinoma (TCGA-COAD) dataset (https://portal.gdc.cancer.gov/projects/TCGA-COAD). The WGCNA package in R3.5.1 was used to create the co-expression network for the common differentially expressed genes (DEGs). Mean connectivity analysis was performed, and a cluster dendrogram was displayed. Then patient age, sex, tumor stage and the expression profiles of RP11-296E3.2, YBX1 and STAT3 were selected as clinical traits to conduct module-trait correlation analysis. Values of |correlation index| > 0.1 and p < 0.05 were considered to indicate significance.

### Exosomal-RNA isolation and detection

HCT116 and RKO cells were cultured separately in three 10 cm dishes. Exosomes were isolated from cell supernatants using ultrahigh-speed centrifugation (200×*g* for 10 min and 2000×*g* for 10 min successively, then 10,000×*g* for 30 min and 100,000×*g* for 90 min) with an Optima XPN-100 Ultracentrifuge (Beckman Coulter, Georgia, USA). Exosomes were isolated from patient serum using an SBI kit (#EXOQ5A-1; System Biosciences, Palo Alto, CA). Total RNA was isolated with TRIzol Reagent. Reverse transcription of RNA was carried out using PrimeScript™RT (#RR036A; TaKaRa, Dalian, China). PCR amplification was performed using UltraSYBR Mixture (#CW0957; CWbio, Jiangsu, China).

### Statistical analysis

For statistical analysis, GraphPad Prism 8 and SPSS 20.0 were employed. Each experiment was performed at least three times independently. The data from the experiments are presented as the means ± standard deviations (SDs). One-way analysis of variance (ANOVA) followed by a Tukey’s post hoc test was used for comparison among three groups, while independent Student’s t test was utilized for comparisons between two groups. Clinical sample data were plotted and are reported as the means. The chi-square test was used to evaluate clinical significance, and Pearson correlation analysis was used to determine correlations between protein staining scores. All P values are reported, and *P < 0.05, **P < 0.01, ***P < 0.001 and ****P < 0.0001 indicate significant differences.

## Results

### Basic information and bioinformatics analyses of RP11-296E3.2

**Fig. 1:**
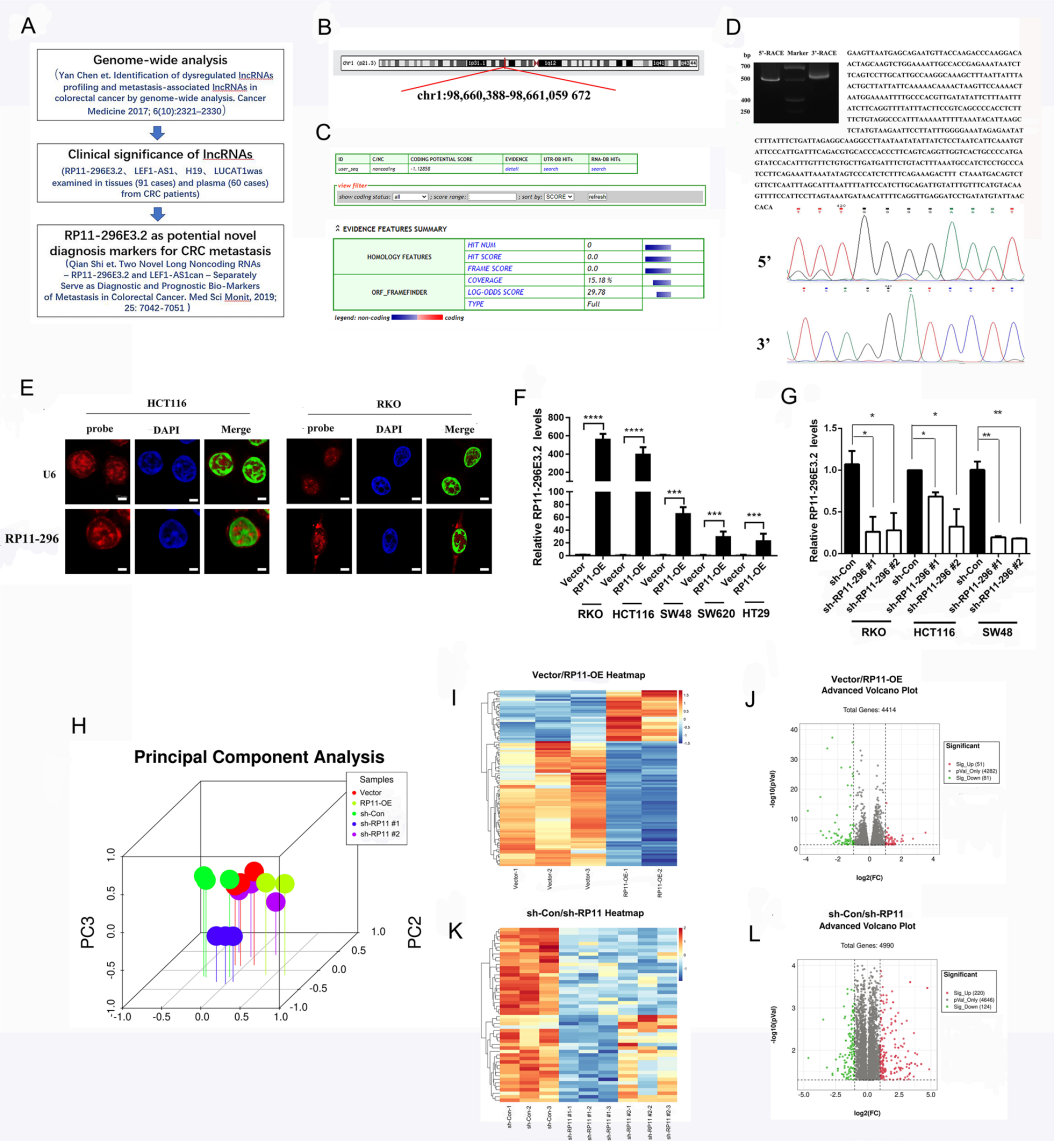
Basic information and bioinformatics analyses of RP11-296E3.2. **A** Schematic illustration of previous clinical studies of RP11-296E3.2 in CRC metastasis. **B** The UCSC Genome Bioinformatics Site (http://genome.ucsc.edu/) showed the location of RP11-296E3.2. **C** Coding Potential Calculator 2 (http://cpc2.gao-lab.org/) showed the protein-coding ability of RP11-296E3.2. **D** The 5′ and 3′ sequences of RP11-296E3.2 were confirmed by RACE and MS. **E** Confocal images showing the RP11-296E3.2 distribution in HCT116 and RKO cells; U6 was used as the nuclear marker. Scale bars: 50 μm. **F**, **G** qRT‒PCR analysis of RP11-296E3.2 expression in RP11-296E3.2-transduced, RP11-296E3.2-silenced and control cells as indicated. The data are presented as the means ± SDs. Statistical significance was assessed using one-way ANOVA followed by Tukey’s test for multiple comparisons. All experiments were performed with at least three biological duplicates (n = 3) for each group. *P < 0.05, **P < 0.01, ***P < 0.001. ****P < 0.0001. **H** 3D PCA showed the differences among the three replicates per group in the RNA-seq results. **I**, **J** Heatmap and volcano plot of SDEGs between RP11-296E3.2-overexpressing and Vector cells. Light red (upregulated) and green (downregulated): genes with |log2FC| > 1 and P_adj_ < 0.05. **K**, **L** Heatmap and volcano plot of SDEGs between sh-RP11-296E3.2 and sh-Con cells. Light red (upregulated) and green (downregulated): genes with |log2FC| > 1 and P_adj_ < 0.05

We previously examined the lncRNAs expression in tissues from CRC patients (91 cases) by qRT-PCR and found that RP11-296E3.2 was closely correlated with CRC metastasis and served as a potential novel diagnostic marker for CRC metastasis (Fig. 1A). Considering the association of lncRNA-RP11-296E3.2 in colorectal metastasis, in this study, we further researched the mechanism of RP11-296E3.2 in colorectal metastasis. First, we used the UCSC Genome Bioinformatics Site and Coding Potential Calculator (CPC) to determine the genomic location and coding potential of RP11-296E3.2. RP11-296E3.2 is located on chromosome 1 and has a total length of 672 bp. The Coding Potential Calculator suggested a low coding potential and low evolutionary conservation consistent with a noncoding RNA (Fig. 1B, C). Then, we identified the 5′ and 3′ ends of RP11-296E3.2 by RACE (Fig. 1D). Next, RNA FISH was performed to determine the localization of RP11-296E3.2, and RP11-296E3.2 was found to be localized mainly in the nucleus in the HCT116 and RKO cell (Fig. 1E). To further study the mechanism by which RP11-296E3.2 contributes to CRC metastasis, several CRC cell lines with stable upregulation or downregulation of RP11-296E3.2 were constructed (Additional file 1: Fig. S1A, B and Additional file 6: Table S3). As shown in Fig. 1F, G, RKO, HCT116 and SW48 cells were the best choices. Subsequently, transcriptome sequencing (Poly(A)-seq) was performed in transduced RKO cells. 3D principal component analysis (PCA) showed small differences among the three replicates per group (Fig. 1H). Heatmaps and Venn diagrams showed that 51 and 81 mRNAs were significantly upregulated and downregulated, respectively, between the vector and RP11-296-OE group (Fig. 1I, J), and that 220 and 124 mRNAs were upregulated and downregulated, respectively, between the sh-Con and sh-RP11-296 groups (Fig. 1K, L).

### RP11-296E3.2 regulates CRC cell proliferation and metastasis in vitro

**Fig. 2:**
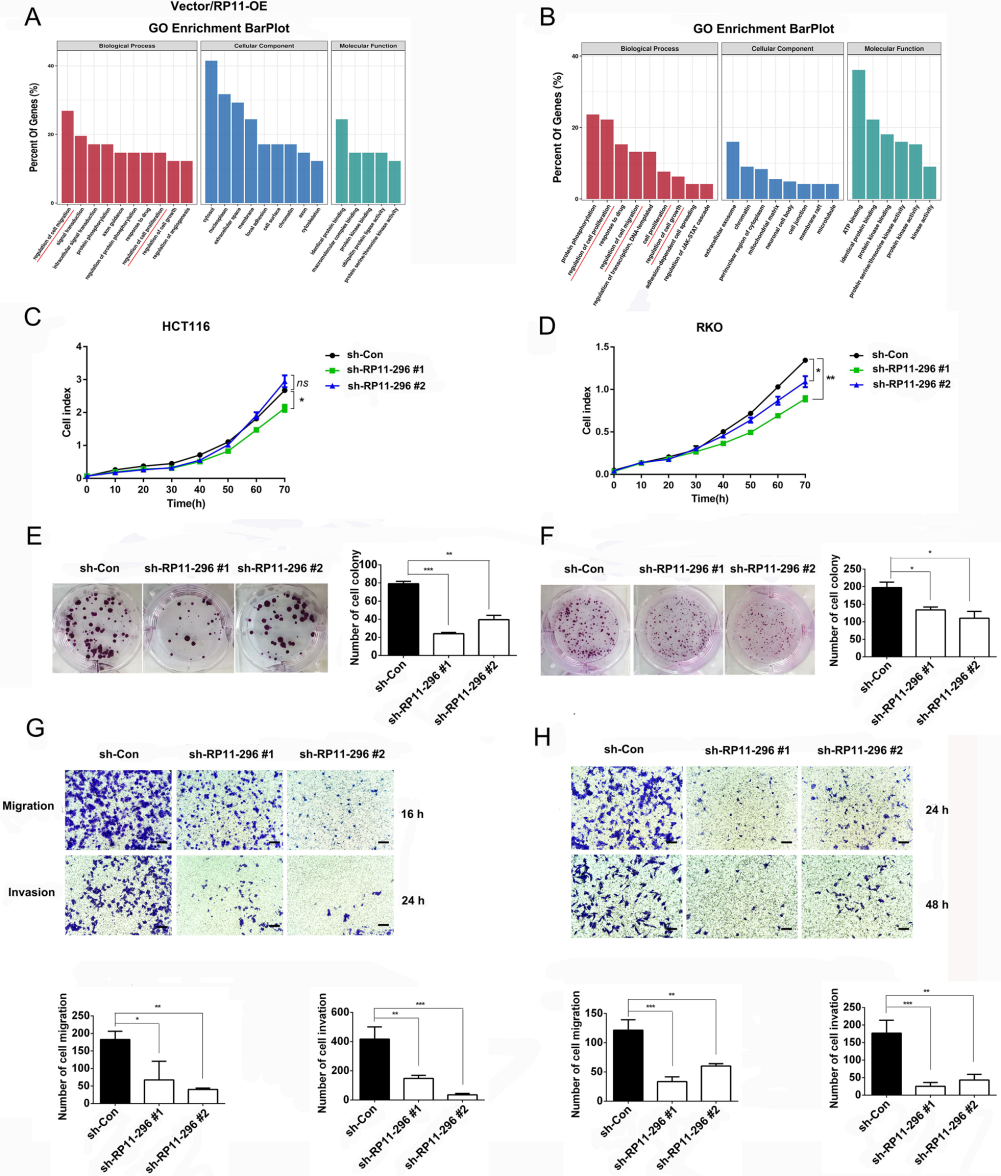
RP11-296E3.2 regulates CRC cell proliferation and metastasis in vitro. **A**, **B** GO enrichment analysis was performed for 132 SDEGs between RP11-296E3.2 overexpressed cells and Vector cells (n = 3 samples per group), and 99 SDEGs overlapping between two sh-RP11-296E3.2 and sh-Con cells (n = 3 samples per group)by online DAVID analysis (https://david.ncifcrf.gov/summary.jsp). **C**, **D** RTCA was performed to evaluate the proliferation of HCT116 and RKO cells transduced with the sh-Con and two sh-RP11-296E3.2 vectors (n = 6 per group). **E**, **F** Colony formation assay showing the effect of RP11-296E3.2 on the growth of RP11-296E3.2-silenced HCT116 and RKO cells relative to the corresponding sh-Con cells. **G**, **H** Representative images of Transwell assays using HCT116 and RKO cells showing cell metastasis and invasion after knockdown of RP11-296E3.2 and a histogram showing the counts of migrated cells. Scale bars: 100 μm. The data are presented as the means ± SDs. Statistical significance was assessed using one-way ANOVA followed by Tukey’s test for multiple comparisons. All experiments were performed with at least three biological duplicates (n = 3) for each group. *P < 0.05, **P < 0.01, ***P < 0.001

GO enrichment analysis was performed with the SDEGs identified by the criteria |log2FC| > 1 and P_adj_ < 0.05 shared between the vector and RP11-296E3.2-OE groups (132 SDEGs) or between the sh-Con and the two sh-RP11-296E3.2 groups (sh-RP11 #1 and sh-RP11 #2 overlapping 99 SDEGs). The results of GO analysis in both paired groups verified that RP11-296E3.2 was closely related to cell migration and proliferation (Fig. 2A, B). RTCA (72 h) and colony formation assays (long-term observation, 2 weeks) were used to evaluate the role of RP11-296E3.2 in CRC cell proliferation (Fig. 2C–F). The proliferation of the two stable sh-RP11-296E3.2 cell lines were significantly slower than that of sh-Con cells in the colony formation assays but not in RTCA, suggesting that determining the effect of RP11-296E3.2 on cell proliferation requires long-term observation. The results of the Transwell assays revealed that the migration and invasion of stably transduced sh-RP11-296E3.2 cells were significantly impaired compared with those of the corresponding control HCT116, RKO and SW48 cells (Fig. 2G, H and Additional file 2: Fig. S2G). In addition, the results of the HCT116 3D tumor spheroid invasion assay and adhesion assay further verified the function of RP11-296E3.2 in invasion and adhesion (Additional file 2: Fig. S2I, J). However, in HCT116 and RKO cells, proliferation, migration, and invasion did not differ between the Vector and stable RP11-296E3.2 overexpression groups (Additional file 2: Fig. S2A–F, H). The reason for this phenomenon is unclear, but it may be related to the continuous export of RP11-296E3.2 from the nucleus (Fig. 1E). Moreover, the expression of metastasis-related proteins was alerted upon RP11296E3.2 knock down (Additional file 3: Fig. S3A).

### RP11-296E3.2 regulates CRC cell proliferation and metastasis in vivo

**Fig. 3:**
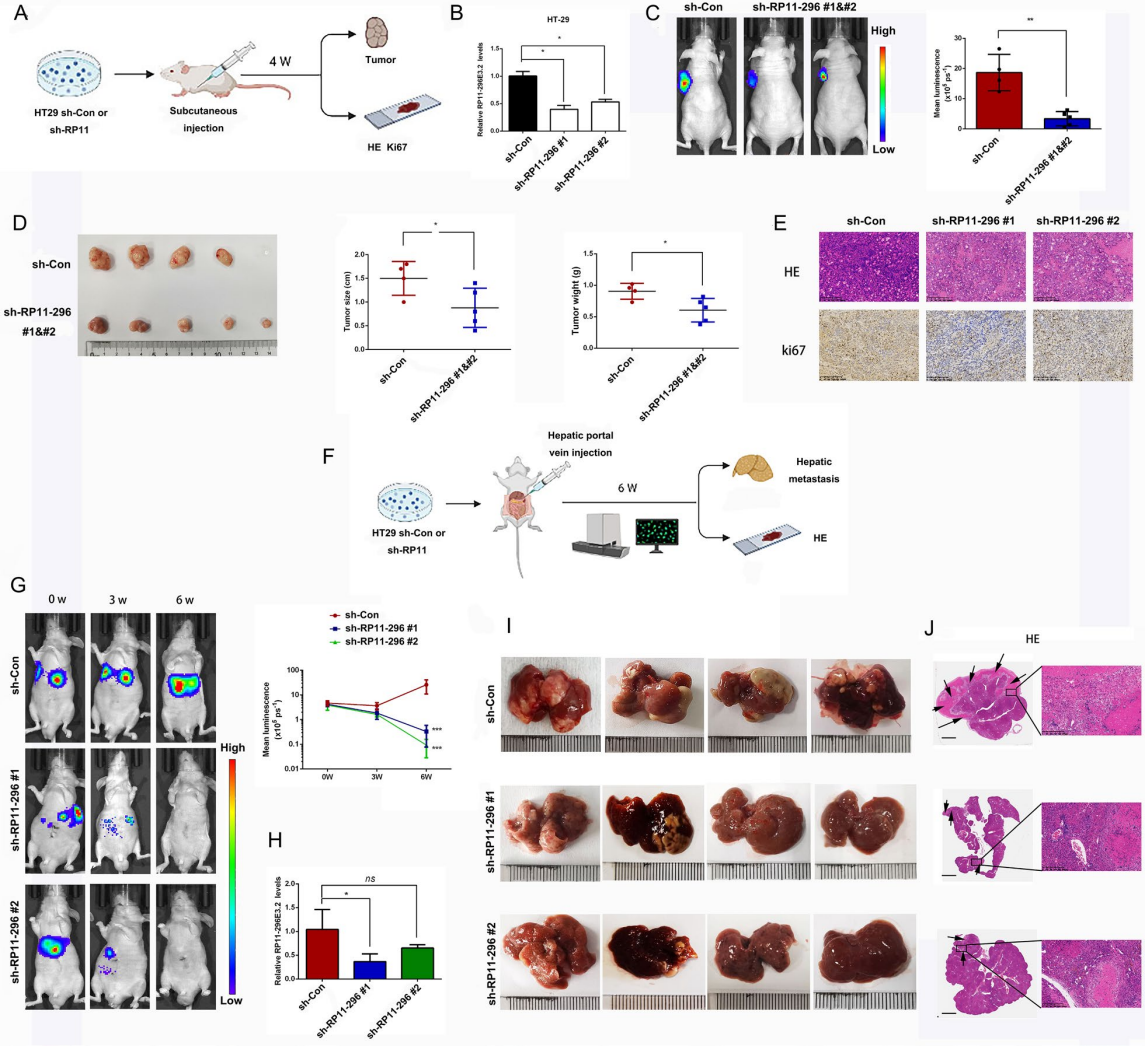
RP11-296E3.2 regulates CRC cell proliferation and metastasis in vivo. **A** Schematic representation of the subcutaneous implantation tumor model. BALB/c nude mice were implanted subcutaneously with HT29 cells (5 × 10^6^), and tumors were allowed to form for 4 weeks. **B** RP11-296E3.2 expression levels in sh-Con and sh-RP11-296E3.2 stably transduced HT29 cells. Knockdown of RP11-296E3.2 inhibited CRC cell proliferation in nude mice. **C** Representative bioluminescence images of subcutaneous tumors, **D** gross morphology of subcutaneous tumors, tumor sizes and tumor weights. The data are presented as the means ± SDs. Statistical significance was assessed using unpaired t test for two comparisons. *P < 0.05, **P < 0.01. **E** HE and Ki67 staining and verification of the RP11-296E3.2 knockdown efficiency in suppressing tumor growth. Scale bars, 200 μm. **F** Schematic depicting the establishment of the liver metastasis xenograft model. The hepatic portal vein of BALB/c nude mice was injected with HT29 cells (5 × 10^6^), and metastases were allowed to form for 6 weeks. **G** Representative bioluminescence images (left) and bioluminescence tracking plots (right). **H** RP11-296E3.2 expression level in liver xenograft tumors in the sh-Con and sh-RP11-296E3.2 groups. **I** Representative gross morphology and **J** HE staining of the livers of mice. Scale bars, 2 mm (left), 200 μm (right). The data are presented as the means ± SDs. Statistical significance was assessed using one-way ANOVA followed by Tukey’s test for multiple comparisons. *P < 0.05, **P < 0.01, ***P < 0.001

To examine the role of RP11-296E3.2 in regulating the proliferation of CRC cells in vivo, HT29 cells stably transduced with the sh-RP11-296E3.2 or the sh-Con vector were inoculated subcutaneously into the axils of BALB/c nude mice (Fig. 3A, B). At week 4 post-inoculation, the mean luminescence intensity in tumors derived from sh-RP11-296E3.2-transduced cells was lower than those of tumors derived from sh-Con-transduced cells (Fig. 3C). The mean weight and volume of tumors derived from sh-RP11-296E3.2-transduced cells were lower than that of tumors derived from sh-Con vector-transduced cells (Fig. 3D). Compared with those in the tumors derived from sh-Con vector-transduced cells, the numbers of HE and Ki-67-positive cells were decreased in the tumors derived from sh-RP11-296E3.2-transduced cells (Fig. 3E). These findings indicated that knockdown of RP11-296E3.2 inhibited tumor growth in vivo.

Next, we examined whether silencing RP11-296E3.2 inhibits metastasis. HT29 cells stably transduced with sh-RP11-296E3.2 or the sh-Con vector were injected into the hepatic portal vein of BALB/c mice to establish a liver metastasis model (Fig. 3F). At 6 weeks after injection, we found that downregulation of RP11-296E3.2 dramatically inhibited liver metastasis compared to the control group, as indicated by bioluminescence and tumor imaging (Fig. 3G, I). HE staining further confirmed that the number of metastatic lesions in the livers of mice inoculated with RP11-296E3.2-knockdown cells were significantly reduced (Fig. 3J). The RP11-296E3.2 expression level in liver tissues was also decreased in the sh-RP11-296E3.2 cell-injected nude mice compared with sh-Con vector cell-injected nude mouse (Fig. 3H). These findings indicated that knockdown of RP11-296E3.2 significantly inhibited CRC metastasis in vivo.

### RP11-296E3.2 regulates the transcription and translation of STAT3 but not JAK2

**Fig. 4:**
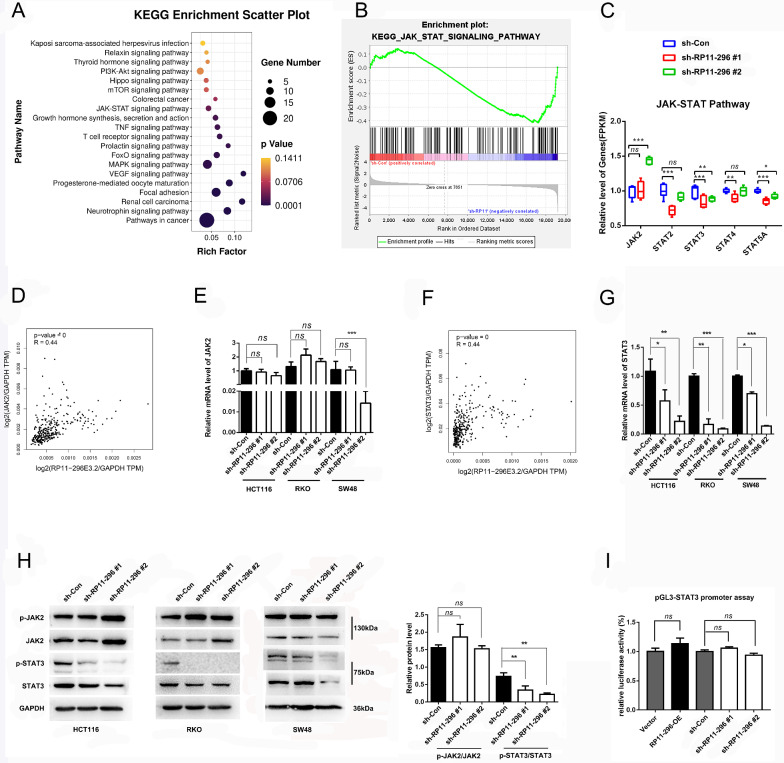
RP11-296E3.2 regulates the transcription and translation of STAT3 but not JAK2. **A** KEGG enrichment analysis of the SDEGs (99 overlapping) between sh-Con- and sh-RP11-296E3.2-transduced RKO cells. P. adjust, hypergeometric test with the Benjamini–Hochberg correction. **B** GSEA of the whole 45,009 genes between in sh-Con- and sh-RP11-296E3.2-transduced RKO cells. The P value was determined by a hypergeometric test. This analysis was performed by GSEA4.3.2 software. **C** mRNA levels of 5 genes in the JAK/STAT3 pathway from the RNA-seq results. **D**, **F** GEPIA2 analysis of the correlations between RP11-296E3.2 and JAK2 or STAT3 expression. **E**, **G** The altered mRNA levels of JAK2 and STAT3 were confirmed by qRT-PCR in HCT116, RKO, and SW48 cells transduced with the indicated shRNAs (n = 3 per group). **H** WB analysis of JAK2, p-JAK2, STAT3 and p-STAT3 in HCT116, RKO and SW48 cells transduced with the indicated shRNAs (n = 3 per group). **I** The STAT3 promoter was evaluated by a luciferase reporter assay in RP11-296E3.2-overexpressing and RP11-296E3.2-silenced cells. The data are presented as the means ± SDs. Statistical significance was assessed using one-way ANOVA followed by Tukey’s test for multiple comparisons. All experiments were performed with at least three biological duplicates (n = 3) for each group. *P < 0.05, **P < 0.01, ***P < 0.001

To characterize the molecular mechanism of RP11-296E3.2 in CRC, a total of 99 SDEGs and the whole genome (45,009 genes) were subjected to KEGG pathway enrichment analysis and GSEA, respectively (Fig. 4A, B and Additional file 3: Fig. S3B). The results of both analyses suggested that RP11-296E3.2 might modulate the JAK/STAT signaling pathway. The results of RNA-seq analysis of JAK/STAT pathway components showed that the mRNA levels of STAT3 and STAT5A were significantly decreased in CRC cell lines with RP11-296E3.2 silencing (Fig. 4C). Moreover, analyses with the GEPIA tool further confirmed that the mRNA levels of JAK2, STAT3 and STAT5A were closely related to that of RP11-296E3.2 (Fig. 4D, F and Additional file 3: Fig. S3D). After knockdown of RP11-296E3.2, the transcript level of STAT3 was significantly decreased (Fig. 4G), but the transcript levels of JAK2, and STAT5A were not significantly different compared with those in the control cells (Fig. 4E and Additional file 3: Fig. S3E). Furthermore, WB analysis revealed that the STAT3 and p-STAT3 protein levels were significantly decreased in cells with stable RP11-296E3.2 silencing, but there was no difference in the JAK2 and p-JAK2 levels (Fig. 4H). In addition, the STAT5A and p-STAT5 protein levels did not differ between sh-Con and sh-RP11-296E3.2 transduced cells (Additional file 3: Fig. S3F). These results indicate that RP11-296E3.2 promotes CRC cell metastasis through activation of STAT3 transcription but not through the classical JAK2/STAT3 signaling pathway. In addition, NCBI BLAST analysis showed that the STAT3 promoter region contained no binding site for the full-length RP11-296E3.2 sequence, and overexpression or silencing of RP11-296E3.2 did not activate the transcriptional activity of the STAT3 promoter (Fig. 4I), suggesting that RP11-296E3.2 regulates STAT3 transcription through other mechanisms.

### RP11-296E3.2 interacts with YBX1 to promote CRC progression

**Fig. 5:**
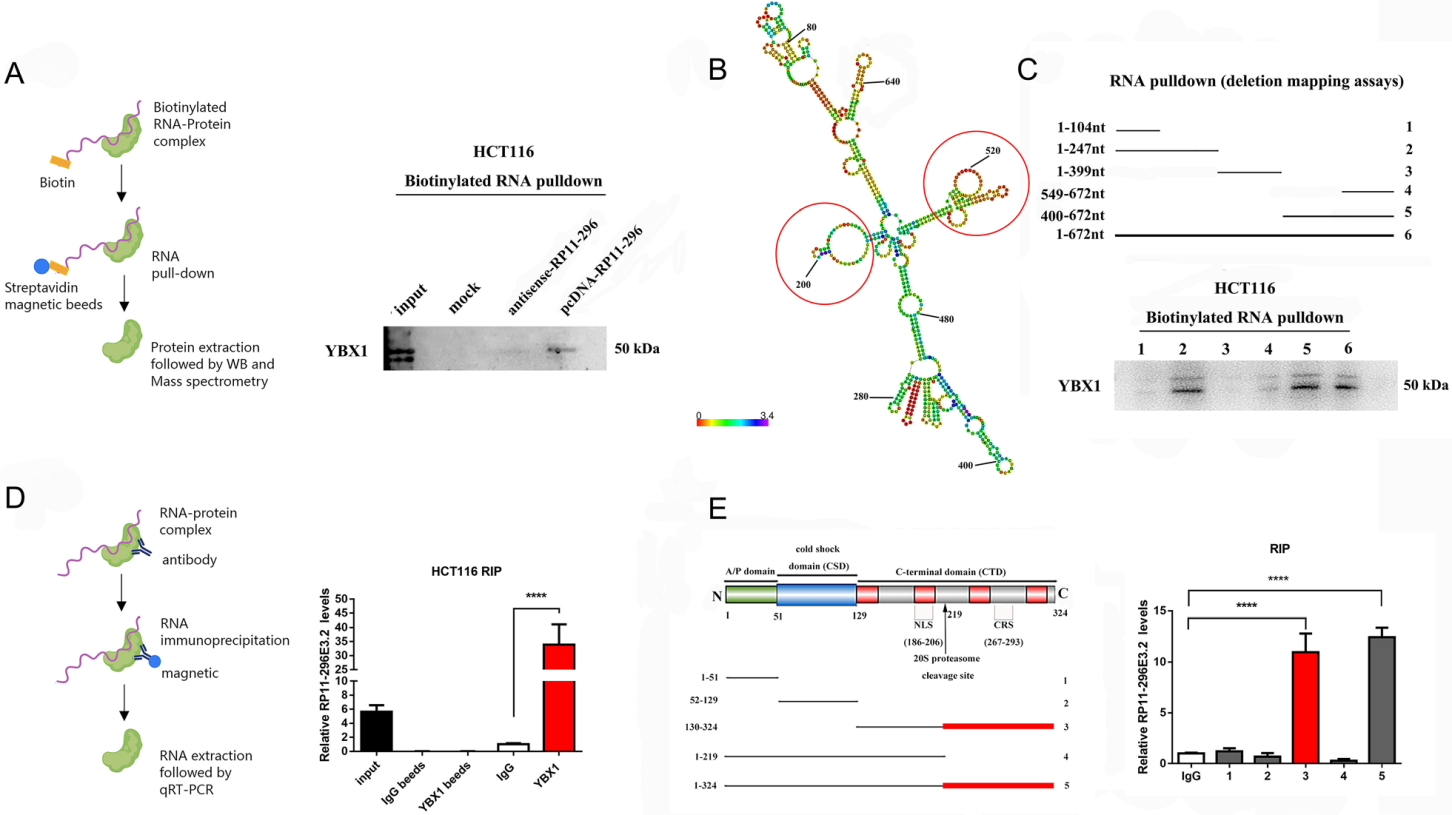
RP11-296E3.2 interacts with YBX1 to promote CRC progression. **A** Model of the RNA pulldown experimental workflow (left panel). The specific association between RP11-296E3.2 and YBX1 in HCT116 cells was validated using an RNA pulldown assay. WB was performed to verify the RNA pulldown assay results. The RP11-296E3.2 antisense sequence was used as a control (right panel). **B** RNAalifold predicted that RP11-296E3.2 contains 5 stable stem‒loop structures. The inset (framed in red) shows the YBX1 binding stem‒loop structures in RP11-296E3.2. **C** RNA pulldown followed by WB analysis was performed with a series of deletion constructs of RP11-296E3.2. **D** Model of the RIP assay workflow (left panel). RIP assays were performed with YBX1. qRT‒PCR analysis revealed that RP11-296E3.2 was significantly enriched in the anti-YBX1 immunoprecipitates relative to the IgG control immunoprecipitates (right panel). **E** RIP assays were performed to quantify the expression of RP11-296E3.2 in cells transfected with YBX1 and its deletion mutants. The data are presented as the means ± SDs. Statistical significance was assessed using one-way ANOVA followed by Tukey’s test for multiple comparisons. ****P < 0.0001

To further identify the specific interacting partners of RP11-296E3.2, in vitro-transcribed RP11-296E3.2 and antisense control sequences were biotinylated and incubated with HCT116 cell lysates to purify RP11-296E3.2 protein complexes (Additional file 4: Fig. S4A). MS was used to identify binding protein(s) (Additional file 4: Fig. S4B), and YBX1, a known RNA-binding protein and transcription factor, was detected in the RP11-296E3.2 protein complex but not in the antisense RP11-296E3.2 protein complex precipitate (Fig. 5A). Moreover, RIP assays showed that YBX1 significantly bound to RP11-296E3.2 compared to other nuclear lncRNAs (for example, ELAVL1) (Fig. 5D and Additional file 4: Fig. S4C, D). To further clarify the binding regions between RP11-296E3.2 and YBX1, we performed RNA pulldown assays using truncated RP11-296E3.2 fragments and cell lysates. Deletion mapping analysis showed that the nt105- to 247 and nt400- to 549 regions of RP11-296E3.2 were indispensable for its interaction with YBX1(Fig. 5C) and formed a stem-loops structure (Fig. 5B). Additionally, a RIP assay was performed to confirm that RP11-296E3.2 specifically bound to YBX1, via binding regions located within amino acids 220–324 in the C-terminal domain (CTD) (Fig. 5E).

### YBX1 is involved in CRC cell proliferation and metastasis

**Fig. 6:**
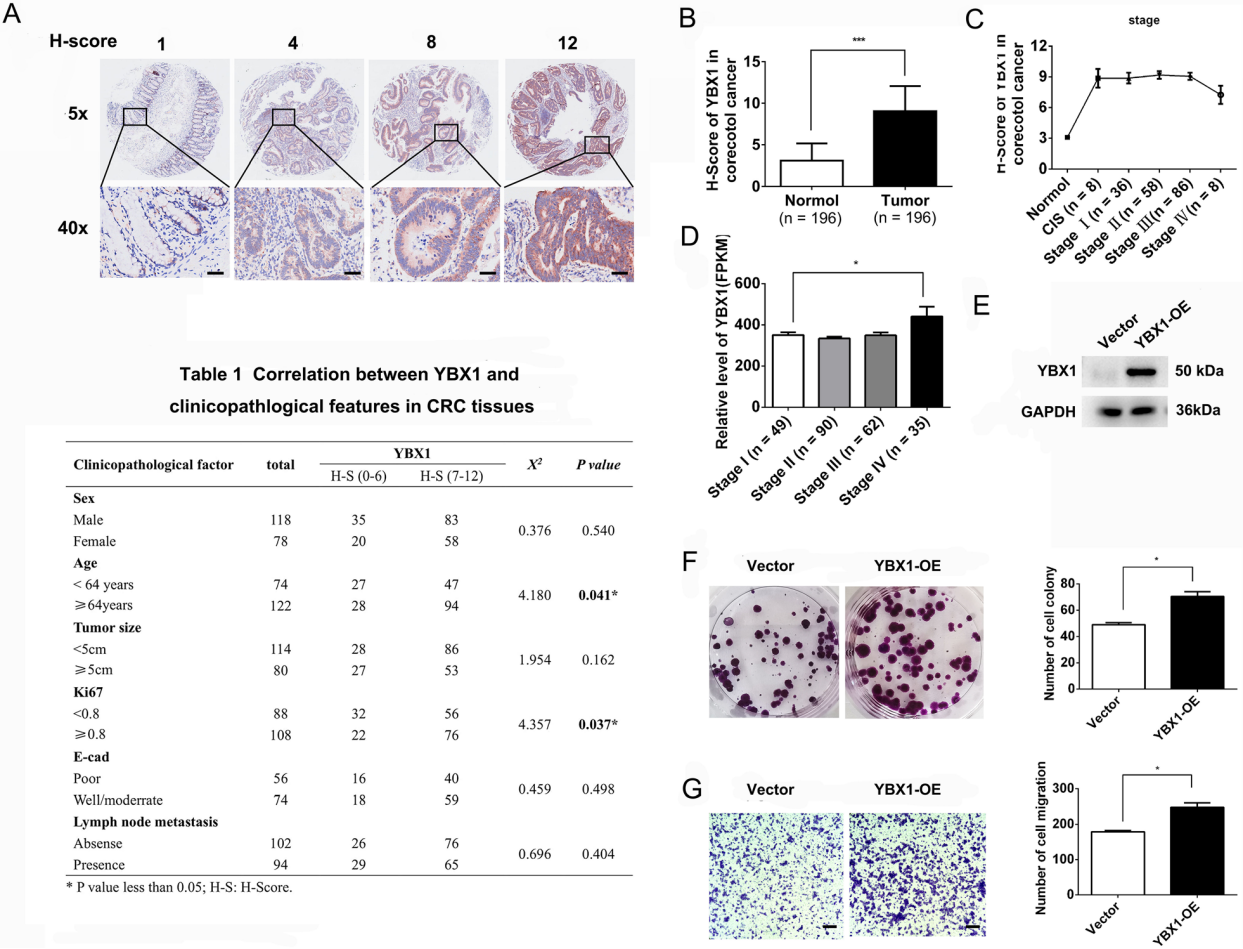
YBX1 is involved in CRC cell proliferation and metastasis. **A**, **B** Images showing signal intensities and the corresponding IHC scores. A total of 195 images were scored. Scale bar: 50 μm (down). **C**, **D** YBX1 expression in CRC patient tumors of different stages (**C** from TAM and **D** from TCGA datasets; Nature 2012). **E** WB analysis of YBX1 expression in HCT116 cells transduced with the YBX1 overexpression vector and control vector. **F**, **G** Colony formation and Transwell assays (scale bars: 100 μm) using HCT116 cells showed the effects of YBX1 on cell proliferation and metastasis. The data are presented as the means ± SDs. Statistical significance was assessed using one-way ANOVA followed by Tukey’s test for multiple comparisons. All experiments were performed with at least three biological duplicates (n = 3) for each group. *P < 0.05, ***P < 0.001

YBX1, a transcriptional activator, is associated with tumorigenesis and tumor development. To examine the role of YBX1 in CRC, we stained a TMA that contained 195 pairs of primary CRC tissues and paracancerous tissues. The images showed that YBX1 expression was significantly increased in the CRC patient TMA (Fig. 6A, B). Each tissue core was scored with the Vectra 2 system, and the scores were further manually curated. Analysis of YBX1 expression in the TMA showed that high YBX1 expression was not correlated with CRC stage (Fig. 6C). Correlation analysis of the IHC score with clinicopathologic parameters showed that YBX1 expression was correlated with Ki67 status (P = 0.037) and age (P = 0.041), but not tumor size (P = 0.162), E-cad status (P = 0.498) or lymph node metastasis status (P = 0.404) (Fig. 6, Table 1). Because there were only 8 cases of stage IV CRC in the TMA, we further used TCGA datasets and found that the level of YBX1 was increased in stage IV CRC (Fig. 6D). Moreover, experimental studies at the cellular level showed that YBX1 can affect the proliferation and metastasis of HCT116 cells (Fig. 6E–G), indicating that YBX1 can affect cell proliferation and distant metastasis in CRC.

### RP11-296E3.2 acts as an important molecular chaperone for YBX1 and promotes CRC cell proliferation and metastasis by activating STAT3 transcription

**Fig. 7:**
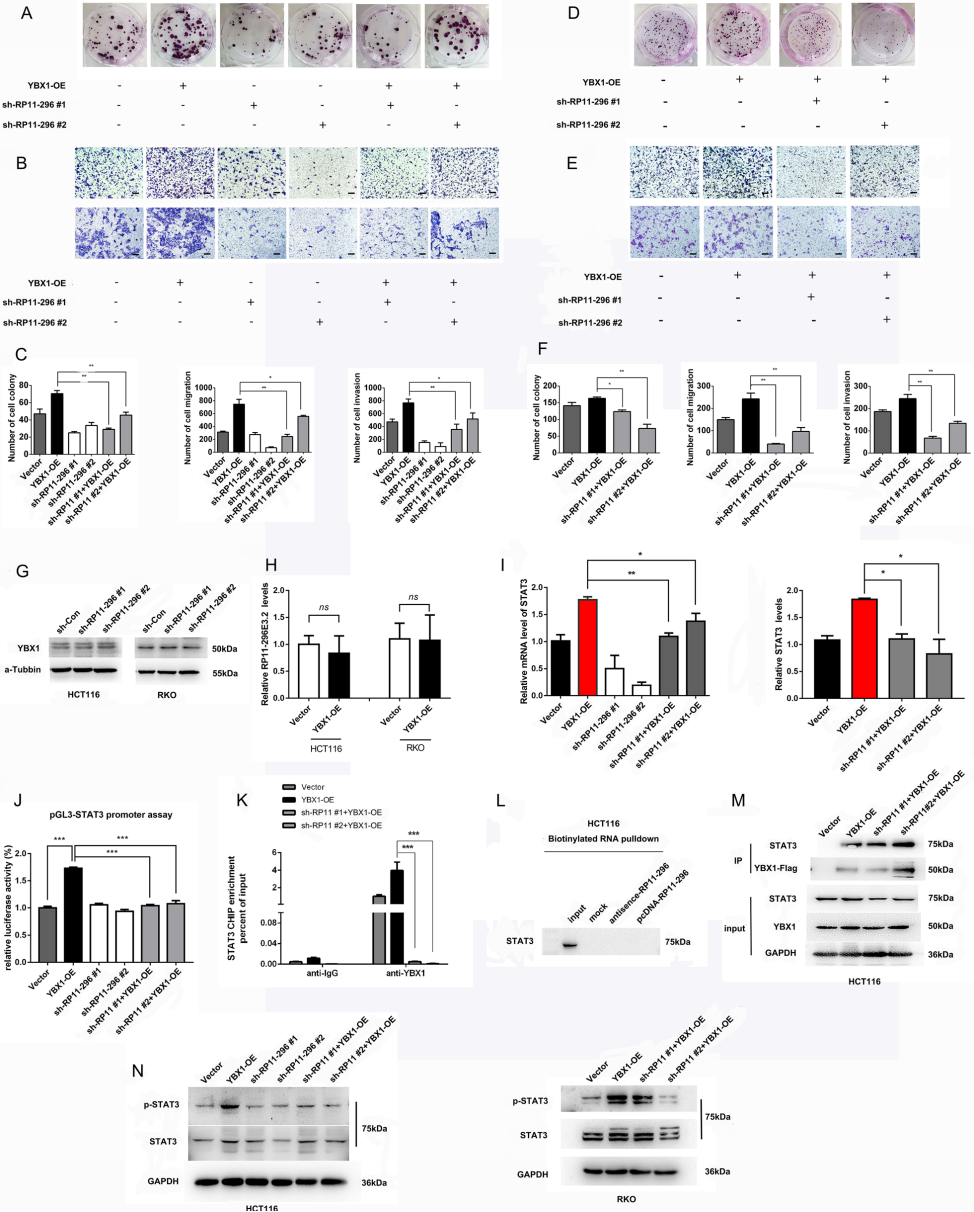
RP11-296E3.2 acts as an important molecular chaperone for YBX1 that functions to promote CRC cell proliferation and metastasis by activating STAT3 transcription. **A** Colony formation and **B** Transwell assays (scale bars: 100 μm) using HCT116 cells showed that RP11-296E3.2 downregulation weakened the effects of YBX1 on cell proliferation, metastasis and invasion. **C** Histogram showing the counts of proliferating and migrated cells. **D** Colony formation and **E** Transwell assays (scale bars: 100 μm) using RKO cells showed that RP11-296E3.2 downregulation weakened the effects of YBX1 on cell proliferation, metastasis and invasion. **F** Histogram showing the counts of proliferating and migrated cells. **G** WB analysis of YBX1 expression in HCT116 cells transduced with the sh-RP11-296E3.2 vector and control vector. **H** qRT‒PCR analysis of RP11-296E3.2 expression in HCT116 cells transduced with the YBX1 overexpression vector and control vector. **I** The altered mRNA levels of STAT3 were confirmed by qRT-PCR in HCT116 and RKO cells transduced with the indicated shRNAs. **J** The STAT3 binding site promoter was subjected to a luciferase reporter assay in HCT116 cells with YBX1 overexpression with or without knockdown of RP11-296E3.2. **K** ChIP assays for the enrichment of YBX1 at the STAT3 promoter in HCT116 cells with YBX1 overexpression with or without knockdown of RP11-296E3.2. **L** The RNA pulldown assay showed that RP11-296E3.2 could not interact with STAT3. **M** The IP assay showed that YBX1 could interact with STAT3 and that silencing of RP11-296E3.2 did not impact the capacity of YBX1 to bind to STAT3. **N** The altered levels of STAT3 and p-STAT3 were confirmed by WB analysis in HCT116 and RKO cells transduced with the indicated shRNAs. The data are presented as the means ± SDs. Statistical significance was assessed using one-way ANOVA followed by Tukey’s test for multiple comparisons. All experiments were performed with at least three biological duplicates (n = 3) for each group. *P < 0.05, **P < 0.01, ***P < 0.001

Given the above results, we examined whether RP11-296E3.2 exerts an important effect on YBX1 to promote CRC cell proliferation and metastasis. As expected, the results of the colony formation assay revealed that overexpression of YBX1 increased the number of colonies formed by HCT116 and RKO cells, while knockdown of RP11-296E3.2 markedly suppressed YBX1-induced CRC cell proliferation. Similarly, the results of Transwell assays revealed that knockdown of RP11-296E3.2 significantly suppressed YBX1-induced cell migration and invasion (Fig. 7A–F).

**Fig. 8:**
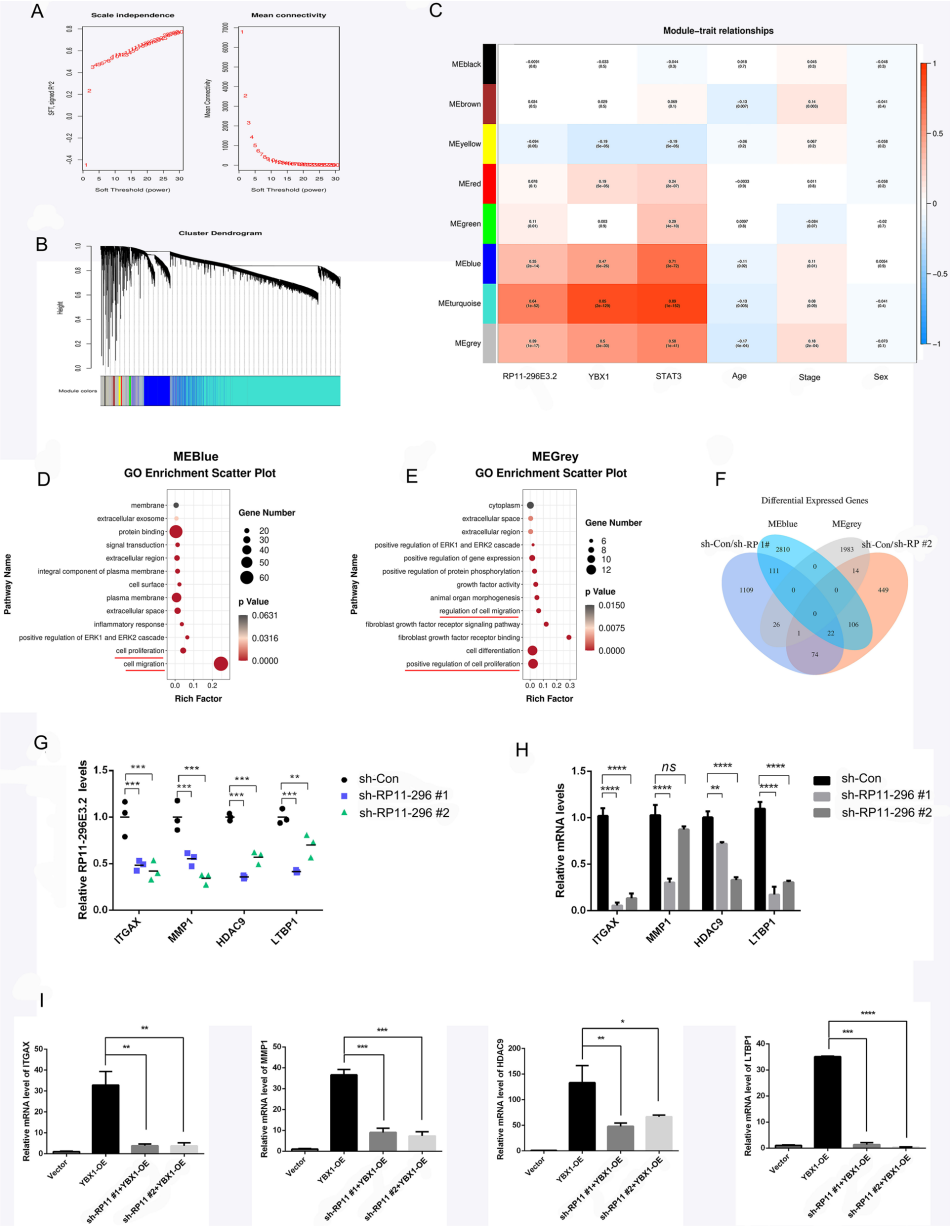
WGCNA and module-trait analysis of the downstream genes closely related to RP11-296E3.2, YBX1, and STAT3. **A** The best soft threshold (β) value was determined depending on scale-free topology and mean connectivity analysis of TCGA data of COAD patients. **B** Hierarchical clustering of the eigengene modules. **C** Correlations between the traits (RP11-296E3.2, YBX1, and STAT3 expression; patient age; tumor stage; and patient sex) and the eigengene modules were analyzed and are shown in the heatmap (R, version 3.5.1). **D**, **E** Top enriched pathways for the significantly correlated modules (MEblue and MEgray). **F** Venn diagram showing the overlapping genes in four comparison sets (MEblue and MEgray module genes and the SDEGs between the sh-Con and sh-RP11-296E3.2 groups). **G** mRNA levels of the 4 overlapping downregulated genes from the RNA-seq results. **H** The altered levels of the 4 overlapping downregulated genes were confirmed by qRT-PCR in HCT116 cells transduced with the indicated shRNAs. **I** Downregulation of RP11-296E3.2 expression weakened the effects of upregulated YBX1 mRNA expression. The data are presented as the means ± SDs. Statistical significance was assessed using one-way ANOVA followed by Tukey’s test for multiple comparisons. All experiments were performed with at least three biological duplicates (n = 3) for each group. *P < 0.05, **P < 0.01, ***P < 0.001

Next, we examined whether RP11-296E3.2 can regulate YBX1 expression, WB showed that RP11-296E3.2 depletion had no effect on the expression of YBX1 (Fig. 7G). Moreover, YBX1 overexpression had no effect on the expression of RP11-296E3.2, but increased the transcript level of STAT3, which was decreased in RP11-296E3.2 silencing HCT116 and RKO cells (Fig. 7H, I). Luciferase activity driven by the STAT3 promoter was increased after overexpression of YBX1, but was significantly decreased upon RP11-296E3.2 silencing (Fig. 7J). To further confirm that RP11-296E3.2 is indispensable for YBX1-mediated activation of STAT3 transcription. ChIP was performed. Silencing RP11-296E3.2 significantly attenuated the promoting effect of YBX1 on the transcriptional activation of STAT3 (Fig. 7K). Taken together, these data suggest that RP11-296E3.2 is indispensable for YBX1-mediated activation of STAT3 transcription and promotes the proliferation and metastasis of CRC cells.

Furthermore, the results of the RNA pulldown assay indicated that RP11-296E3.2 could not interact with STAT3, and the results of the IP assay showed that silencing RP11-296E3.2 could not impair the binding of YBX1 and STAT3 (Fig. 7L, M), suggesting that the increase in the p-STAT3 level is affected only by changes in the STAT3 status (Fig. 7N). Moreover, we further investigated whether RP11-296E3.2 affects the cytoplasmic or nuclear localization of YBX1. The results of IP and WB revealed that YBX1 was distributed mainly in the cytoplasm, with a small amount in the nucleus, however, phosphorylated YBX1 was localized only in the nucleus. Knockdown of RP11-296E3.2 induced phosphorylation YBX1 accumulated in nuclear (Additional file 5: Fig. S5A, B), suggesting that RP11-296E3.2 can inhibit YBX1 phosphorylation and that Phospho-YBX1 may perform other biological functions.

### WGCNA and module-trait analysis of the downstream genes closely related to RP11-296E3.2, YBX1 and STAT3

We next sought to ascertain the mechanism of RP11-296E3.2/YBX1-driven colorectal tumorigenesis and to determine the downstream genes associated with RP11-296E3.2, YBX1, STAT3, and clinicopathologic features of patients. We downloaded TCGA date from 454 cases of COAD patients. Samples were subjected to independence and mean connectivity analysis, and the best soft threshold (β) value was automatically determined to be 8 (Fig. 8A). Then, a total of 8 gene modules are obtained and were presented using different colors (Fig. 8B). Next, the expression levels of the 3 genes, the patient age, the patient sex, and the tissue malignancy grade represented by the pathological subtype were chosen as the traits, and the relationships of these traits with the abovementioned 8 modules were analyzed (Fig. 8C). Notably, the expression levels of all 3 genes and CRC tumor stage were closely related to the MEblue module (RP11-396E3.2, cor = 0.35, P = 2e−14; YBX1, cor = 0.47, P = 6e−26; STAT3, cor = 0.71, P = 3e−72; stage, cor = 0.11, P = 0.01), and MEgray module (RP11-396E3.2, cor = 0.39, P = 1e−17; YBX1, cor = 0.5, P = 3e−30; STAT3, cor = 0.58, P = 1e−41; stage, cor = 0.18, P = 2e−04). GO enrichment analysis confirmed that MEblue and MEblack module genes were closely associated with CRC tumor metastasis and proliferation (Fig. 8D, E). Additionally, we identified 22 genes overlapping between the set of SDEGs in sh-Con cells compared with sh-RP11-296E3.2 cells. and the MEblue module genes. One gene was common to the set of SDEGs in sh-Con cells compared with sh-RP11-296E3.2 cells. and the MEgray module genes (Fig. 8F). The overlapping significantly downregulated genes were identified for further research (Fig. 8G, H). RT-PCR analysis demonstrated that RP11-296E3.2 attenuated the promoting effect of YBX1 on the expression of those downregulated genes (Fig. 8I).

### RP11-296E3.2 is enriched in exosomes and closely correlated with YBX1

**Fig. 9:**
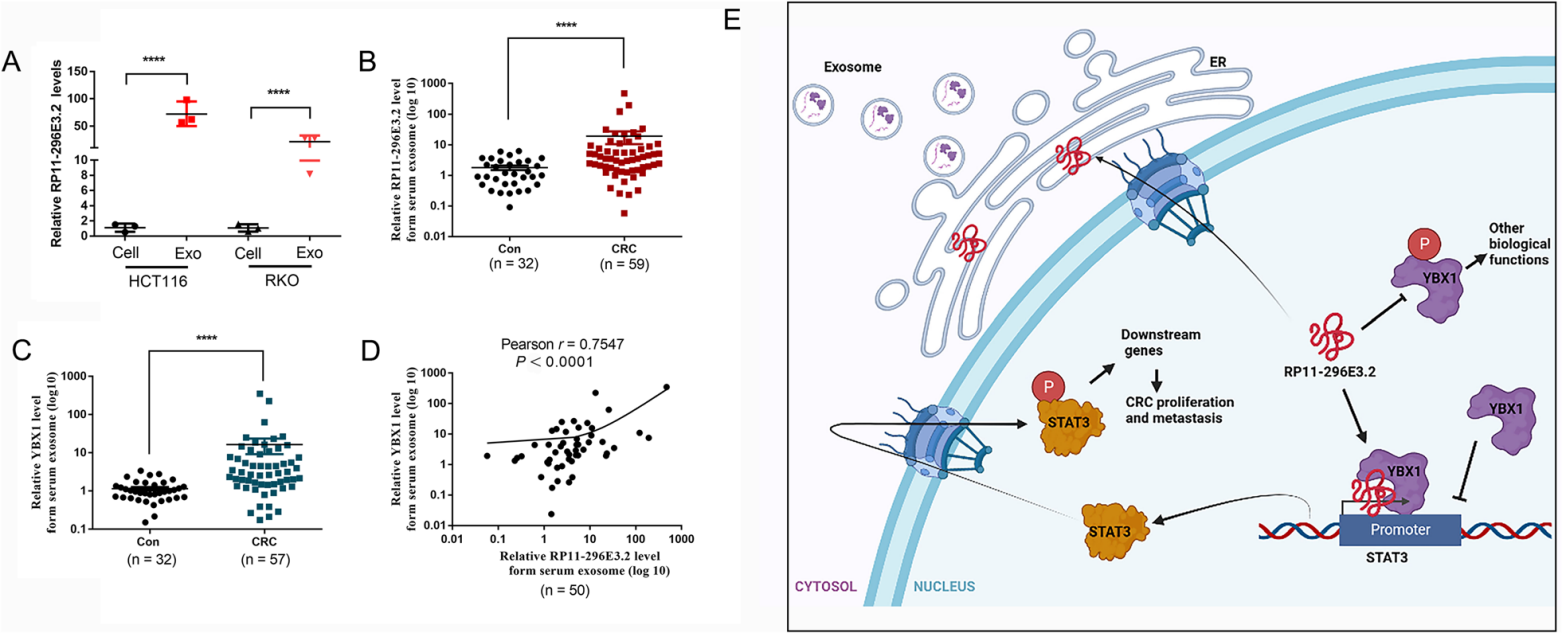
RP11-296E3.2 is enriched in exosomes and closely correlated with YBX1. **A** Expression level of RP11-296E3.2 in HCT116 and RKO cells and cell supernatant exosomes (n = 3 per group). **B** Expression level of RP11-296E3.2 in patient and healthy control serum exosomes. **C** Expression level of YBX1 in patient and healthy control serum exosomes. The data are presented as the means ± SDs. Statistical significance was assessed using unpaired t test for two comparisons. ****P < 0.0001. **D** Correlation of the abundances of RP11-296E3.2 and YBX1 in serum exosomes. The correlation between RP11-296E3.2 and YBX1 was using Two-tailed Pearson correlation analysis. **E** Proposed model in which RP11-296E3.2 mediates CRC cell proliferation and migration of CRC progression

We hypothesized that RP11-296E3.2 may be continually packaged into exosomes to perform its function, based on the FISH results (RP11-296E3.2 was also abundant in the perinuclear region) (Fig. 1E). RP11-296E3.2 was abundant in exosomes isolated from HCT116 and RKO cell supernatants (Fig. 9A), as expected. RP11-296E3.2 and YBX1 were highly abundant in the serum exosomes of CRC patients compared to healthy controls and had a strong mutual association (Fig. 9B–D). Determining the function and mechanism of exosomal-RP11-296E3.2 requires in-depth study of large samples.

In summary, these data suggest that the RP11-296E3.2-YBX1-STAT3 axis is involved in human CRC development, and they provide promising diagnostic biomarkers and therapeutic targets for CRC (Fig. 9E).

## Discussion

RP11-296E3.2, a new lncRNA, that exists only in humans, was found to be strongly associated with mCRC in our prior clinical investigation [7]. Since RP11-296E3.2 has very high expression in metastatic and drug-resistant CRC patient tissue, it may be a viable new marker for the prognosis and diagnosis of mCRC. In the present study, we revealed that inhibition of RP11-296E3.2 expression significantly reduced the ability of CRC cell to form tumors and metastasize both in vitro and in vivo. Mechanistically, RP11-296E3.2 regulated STAT3 expression by recruiting YBX1 to the STAT3 promoter and then activating STAT3 transcription. However, upon knockdown of RP11-296E3.2, the transcription of STAT3 was subsequently inhibited even though YBX1 was upregulated. Together, these results suggest that RP11-296E3.2 is an independent molecular chaperone for YBX1 that functions to promote CRC cell proliferation and metastasis by activating the transcription and phosphorylation of STAT3.

LncRNAs, are present in both the cytoplasm and nucleus and are involved in numerous biological processes, including gene regulation, development, and nuclear organization [10]. Many studies on individual lncRNAs have identified their molecular functions as decoys, recruiters and scaffolds, which are mediated through interactions with proteins and the subsequent formation of ribonucleoproteins [11]. Although research on RP11-296E3.2 is scarce, there are several lncRNAs whose functional mechanisms are similar to those of RP11-296E3.2. For instance, metastasis associated lung adenocarcinoma transcript 1 (MALAT1) binds to splicing factor proline and glutamine rich (SFPQ) and dissociates the SFPQ/PTBP2 dimer to release free polypyrimidine tract binding protein 2(PTBP2), which increases the translation of RUNX family transcription factor 2 (RUNX2) [12]. Moreover, higher RUNX2 expression levels were found in recurrent CRC tumors [13], and were directly related to TNM stage, metastasis, and CRC patient survival. In a recent study, Xia et al. also found that lnc-CTHCC can bind to heterogeneous nuclear ribonucleoprotein K (hnRNP K), and that this interaction promoted hepatocellular tumorigenesis and progression by activating Yes1 associated transcriptional regulator (YAP1) transcription [14]. In our study, JAK2/STAT3 was first recognized as the primary regulatory pathway of RP11-296E3.2. These findings suggest that RP11-296E3.2 may affect the transcription and translation of STAT3 but not of JAK2. Strangely, RP11-296E3.2 did not impact STAT3 promoter activity, indicating that STAT3 promoter activity may have additional regulatory mechanisms.

YBX1, is a well-known oncogenic transcription factor, multifunctional RNA-/DNA-binding protein, and an interaction node in the noncoding transcriptome, exosomal signaling, and cytoplasmic granule signaling [15]. YBX1, containing a classical cold shock domain (CSD), an Ala/Pro-rich (A/P) domain, and a long CTD, plays an important role in tumorigenesis by binding to promoters and promoting the transcription of several genes, including forkhead box A1 (FOXA1) [16], notch receptor 3 (NOTCH3) [17], and so on [18]. The existence of the YBX1/STAT3 pathway is a novel finding that we are the first to discover and confirm, and this pathway depends on RP11-296E3.2. Our results showed that RP11-296E3.2 specifically bound to the region within amino acids 220–324 in the YBX1 CTD. Aberrant RP11-296E3.2 expression impairs YBX1 function, and leads to reduced STAT3 transcription, translation and phosphorylation, thus inhibiting the proliferation and metastasis of CRC cells. Moreover, YBX1 expression is strongly correlated with CRC cell proliferation, and it can be a marker of distant organ metastasis but not lymphatic metastasis. This finding is supported by the observation that YBX1 expression was dramatically increased in the tissues of CRC patients with stage IV, but not in those of patients with stage III or stage II, according to our clinical research and TCGA analysis. There are few studies on phospho-YBX1, however, Yuchen Bai et al. recently discovered that YBX1 was phosphorylated and translocated to the nucleus as a result of oncogenic PI3K/mTOR signaling, an event that promotes cell growth. Phospho-YBX1 is a good prognostic indicator in patients with heterogeneous head and neck cancer (HNC) [19]. We found that knockdown of RP11-296E3.2 dramatically boosted the level of phospho-YBX1 in the nucleus, although its roles require more investigation.

Why does overexpression of RP11-296E3.2 not impact the growth and metastasis of CRC? First, according to the results of the rescue experiment, for RP11-296E3.2 to be effective, it must interact YBX1; without YBX1, RP11-296E3.2 is ineffective. Alternatively, RP11-296E3.2 may be continuously generated, released into the endoplasmic reticulum (ER), and then packaged into exosomes to participate in distant CRC metastasis. This explanation is supported by the results of our FISH and preliminary exosome-related assays. This mechanism is similar to that of taurine up-regulated 1 (TUG1), which was initially thought to have a role in the nucleus but was later discovered to be a highly abundant ER enriched lncRNA [20]. Exosomal-RP11-296E3.2 is therefore a unique molecule that needs more research.

In conclusion, our findings showed that RP11-296E3.2 is a conserved, highly expressed lncRNA in mCRC. RP11-296E3.2 plays an oncogenic role in colorectal tumorigenesis and metastasis. Mechanistically, by activating STAT3 transcription and phosphorylation, RP11-296E3.2 functions as a crucial molecular chaperone for YBX1 that promotes CRC spread and proliferation. Similarly, RP11-296E3.2 plays a crucial role in the oncogenic YBX1/STAT3 axis that drives CRC metastasis, suggesting that it could be used as a prognostic marker and a therapeutic target in mCRC.

## Supplementary Information

**Additional file 1: Figure S1.** The interference segment position of two sh-RP11-296E3.2s.

**Additional file 2: Figure S2.** The effects of RP11-296E3.2 on the proliferation, metastasis and invasion of colorectal cells. A–F, HCT116 and RKO cells were transfected with Vector and RP11-296E3.2. (A, B) RTCA (4 d), (B, E) A colony formation assay (2 weeks) and (C, F) Transwell assays (16 h and 24 h) were performed to evaluate cell proliferation, metastasis and invasion (Scale bars: 100 μm). (G) SW48 cells were transfected with sh-Con or sh-RP11-296E3.2 #1 and #2 for 48 h, and Transwell assays were performed to evaluate cell metastasis (Scale bars: 100 μm). (H, I) HCT116 cells were transfected with indicated vector and resuspended in Matrigel (1:2) to allow the formation of tumor spheroids. 3D invasion of spheroids was monitored for up to 6 d using an imaging cytometer (Scale bars: 500 μm). (J) HCT116 cells were transfected with the indicated vector and incubated for 3 h, and the number of adhered cells was determined with an imaging cytometer (Scale bars: 50 μm).

**Additional file 3: Figure S3.** Regulatory mechanism of RP11-296E3.2 on the MAPK and STAT5A pathways. (A) Metastasis-related protein expression in RP11-296E3.2-downregulated HCT116 cells. (B) Genome-wide GSEA of sh-Con- and sh-RP11-296E3.2-transducted RKO cells. P values were determined by a hypergeometric test. (C) mRNA levels of 4 genes in the MAPK pathway, as determined by RNA-seq analysis. (D) GEPIA2 analysis of the correlation between RP11-296E3.2 and STAT5A. (E) The altered mRNA levels of STAT5A in HCT116 and HT29 cells transfected with the indicated siRNAs were confirmed by qRT-PCR. (F) WB analysis of STAT5A and p-STAT5A in HCT116 and HT29 cells transfected with the indicated siRNAs.

**Additional file 4: Figure S4.** RNA pulldown and RIP assays of RP11-296E3.2 and the effect of YBX1 on CRC stage. (A) An RNA pulldown assay was performed using RP11-296E3.2 sense and antisense RNAs in HCT116 cells, followed by silver staining. The red arrow indicates YBX1. (B) Representative ELAVL1 and YBX1 peptides identified by MS. (C) WB analysis was performed to verify the results of the ELAVL1 and RACK1 RNA pulldown assay. The RP11-296E3.2 antisense RNA was used as a control. (D) RIP experiments were performed with ELAVL1.

**Additional file 5: Figure S5.** Knockdown of RP11-296E3.2 increased the level of nuclear p-YBX1. (A) WB analysis showed that knockdown of RP11-296E3.2 induced an increase in nuclear p-YBX1 in HCT116 and RKO cells. (B) Confocal microscopy analysis of YBX1 in RKO cells. Knockdown of RP11-296E3.2 induced nuclear accumulation of p-YBX1 (Scale bars: 40 μm).

**Additional file 6: Table S1.** Primers for qRT-PCR. **Table S2.** Primers for vectors construction. **Table S3.** Antibodies.

**Additional file 7.** Complete results of the GSEA.

## Data Availability

The data in the current study are available from the first author upon request.

## Abbreviations

CRC: Colorectal cancer
mCRC: Metastatic colorectal cancer
TCGA: Cancer Genome Atlas program
FISH: Fluorescence in situ hybridization
RIP: RNA-interacting protein immunoprecipitation
TMA: Tissue microarray
ChIP: Chromatin immunoprecipitation
RACE: Rapid amplification of cDNA ends
PCA: 3D principal component analysis
GO: Gene ontology
KEGG: Kyoto Encyclopedia of Genes and Genomes
GSEA: Gene set enrichment analysis
DEGs: Differentially expressed genes
SDEGs: Significant differentially expressed genes
ULA: Ultralow attachment
RNA-seq: RNA sequencing
qRT-PCR: Real-time quantitative polymerase chain reaction
WGCNA: Weighted correlation network analysis
CPC: Coding Potential Calculator
shRNA: Short hairpin RNA
lncRNA: Long noncoding RNA
YBX1: Y-Box Binding Protein 1
STAT3: Signal transducer and activator of transcription 3
CCAT1: Colon cancer-associated transcript 1
CSD: Classical cold shock domain
A/P: Ala/Pro-rich
CTD: C-terminal domain
HNC: Heterogeneous head and neck cancer
ER: Endoplasmic reticulum
MALAT1: Metastasis associated lung adenocarcinoma transcript 1
SFPQ: Splicing factor proline and glutamine rich
PTBP2: Polypyrimidine tract binding protein 2
RUNX2: RUNX family transcription factor 2
hnRNP K: Heterogeneous nuclear ribonucleoprotein K
YAP1: Yes1 associated transcriptional regulator
FOXA1: Forkhead box A1
NOTCH3: Notch receptor 3
TUG1: Taurine up-regulated 1
