# Supplementary figures and images for: RP11-296E3.2 acts as an important molecular chaperone for YBX1 and promotes colorectal cancer proliferation and metastasis by activating STAT3
Source: J Transl Med. 2023 Jun 27;21:418. doi: 10.1186/s12967-023-04267-4 (PMC10303830; doi:10.1186/s12967-023-04267-4)

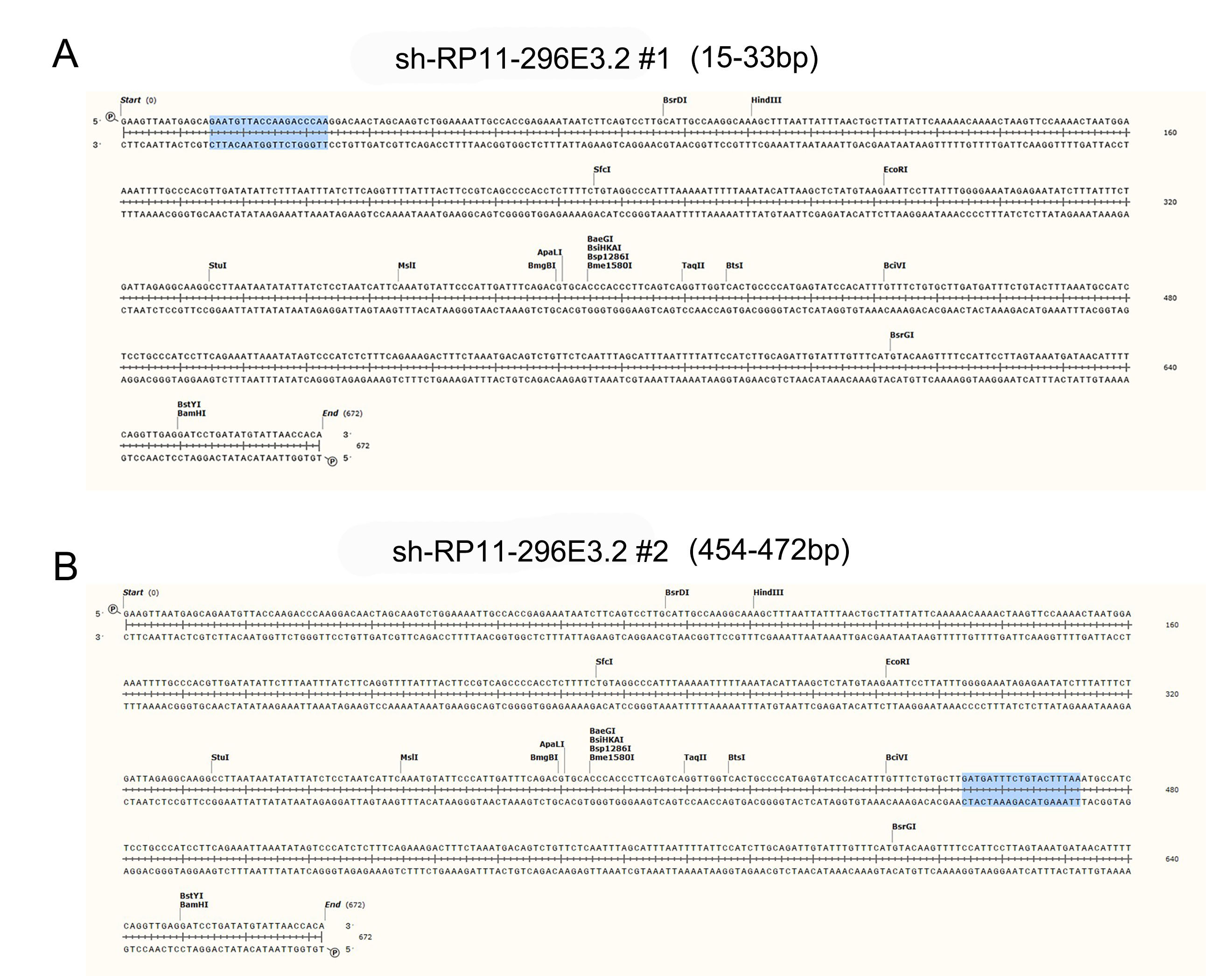

Supplement: Supplementary file 1 — Additional file 1: Figure S1. The interference segment position of two sh-RP11-296E3.2s. [file 12967_2023_4267_MOESM1_ESM.jpg]

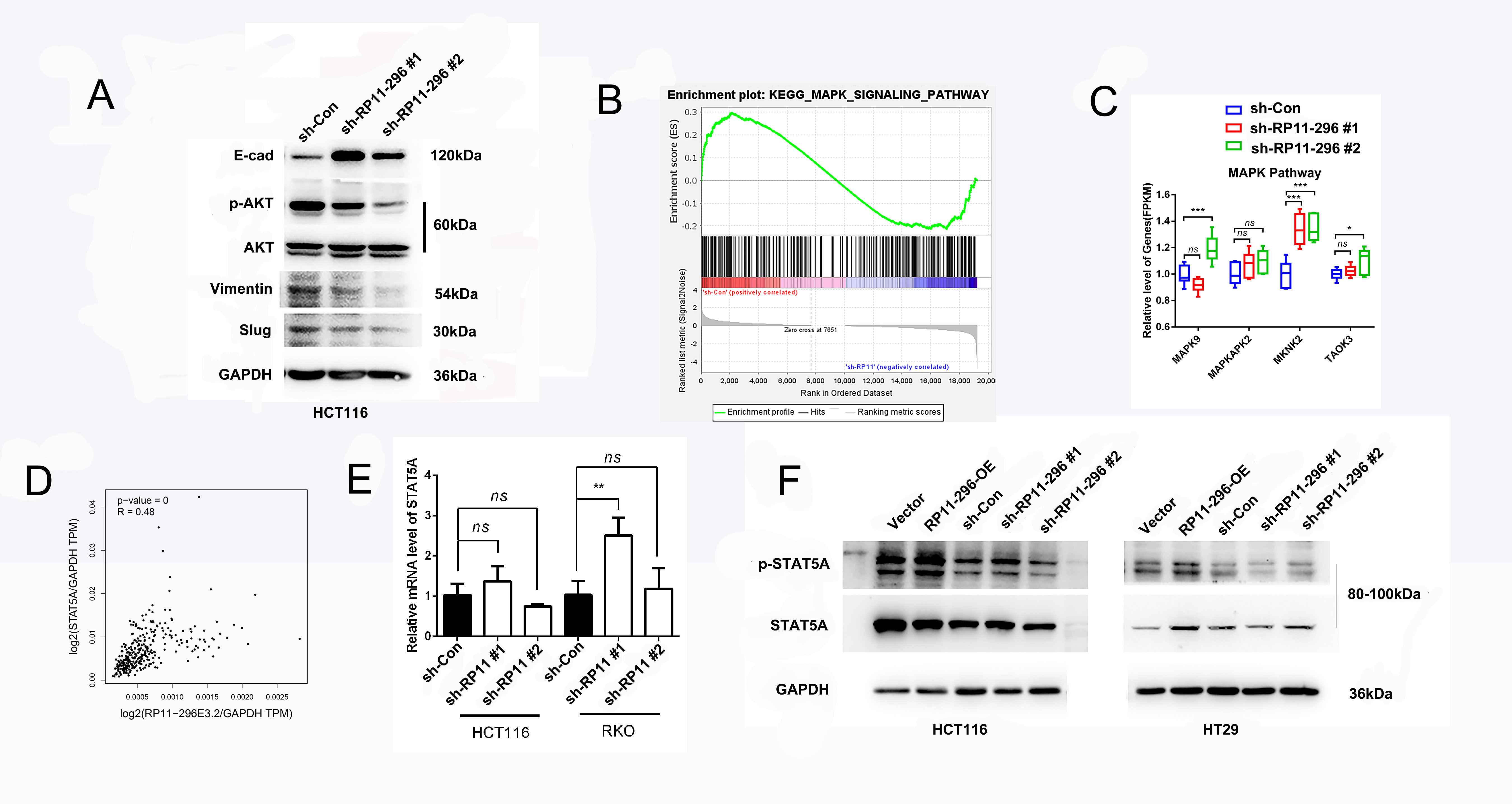

Supplement: Supplementary file 3 — Additional file 3: Figure S3. Regulatory mechanism of RP11-296E3.2 on the MAPK and STAT5A pathways. (A) Metastasis-related protein expression in RP11-296E3.2-downregulated HCT116 cells. (B) Genome-wide GSEA of sh-Con- and sh-RP11-296E3.2-transducted RKO cells. P values were determined by a hypergeometric test. (C) mRNA levels of 4 genes in the MAPK pathway, as determined by RNA-seq analysis. (D) GEPIA2 analysis of the correlation between RP11-296E3.2 and STAT5A. (E) The altered mRNA levels of STAT5A in HCT116 and HT29 cells transfected with the indicated siRNAs were confirmed by qRT-PCR. (F) WB analysis of STAT5A and p-STAT5A in HCT116 and HT29 cells transfected with the indicated siRNAs. [file 12967_2023_4267_MOESM3_ESM.jpg]

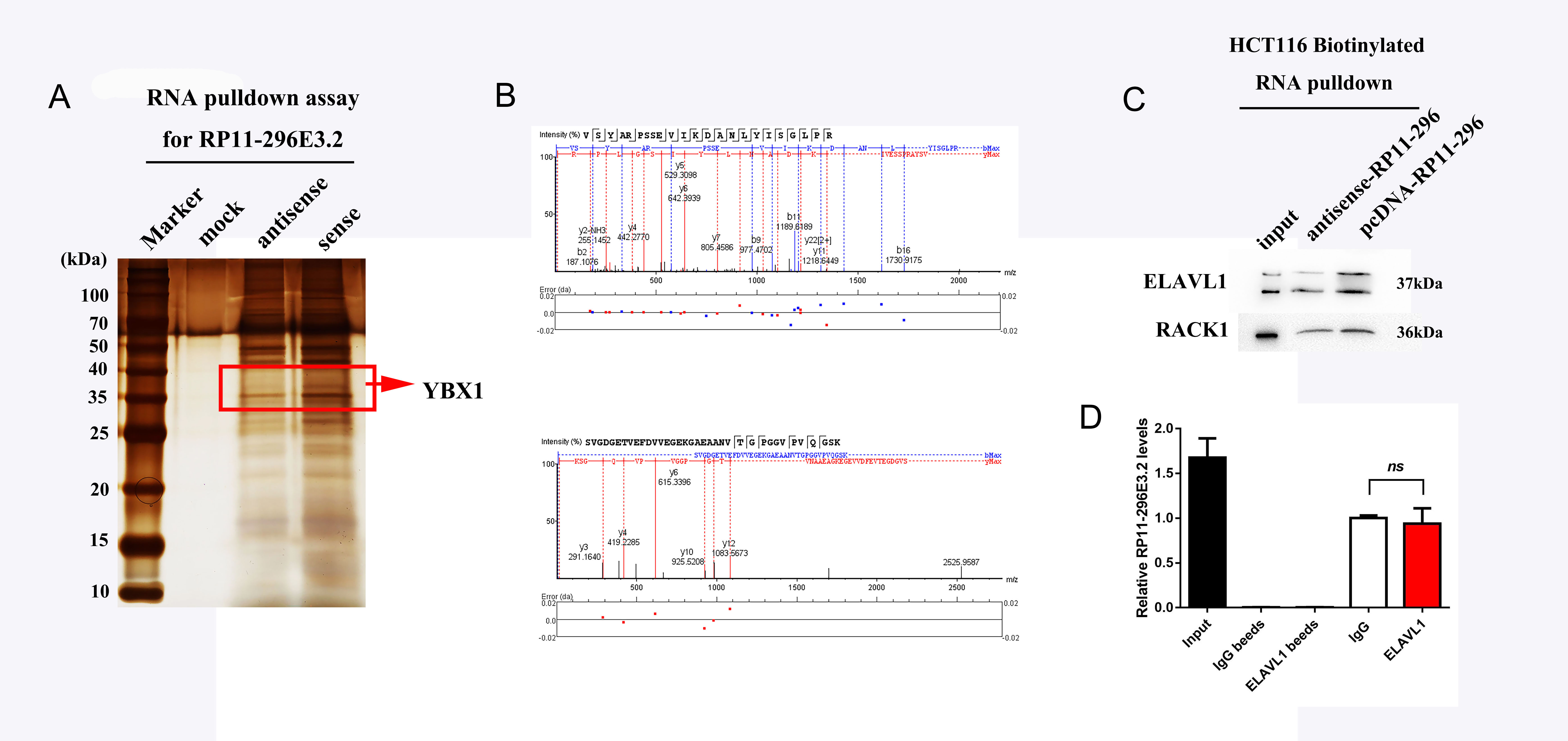

Supplement: Supplementary file 4 — Additional file 4: Figure S4. RNA pulldown and RIP assays of RP11-296E3.2 and the effect of YBX1 on CRC stage. (A) An RNA pulldown assay was performed using RP11-296E3.2 sense and antisense RNAs in HCT116 cells, followed by silver staining. The red arrow indicates YBX1. (B) Representative ELAVL1 and YBX1 peptides identified by MS. (C) WB analysis was performed to verify the results of the ELAVL1 and RACK1 RNA pulldown assay. The RP11-296E3.2 antisense RNA was used as a control. (D) RIP experiments were performed with ELAVL1. [file 12967_2023_4267_MOESM4_ESM.jpg]

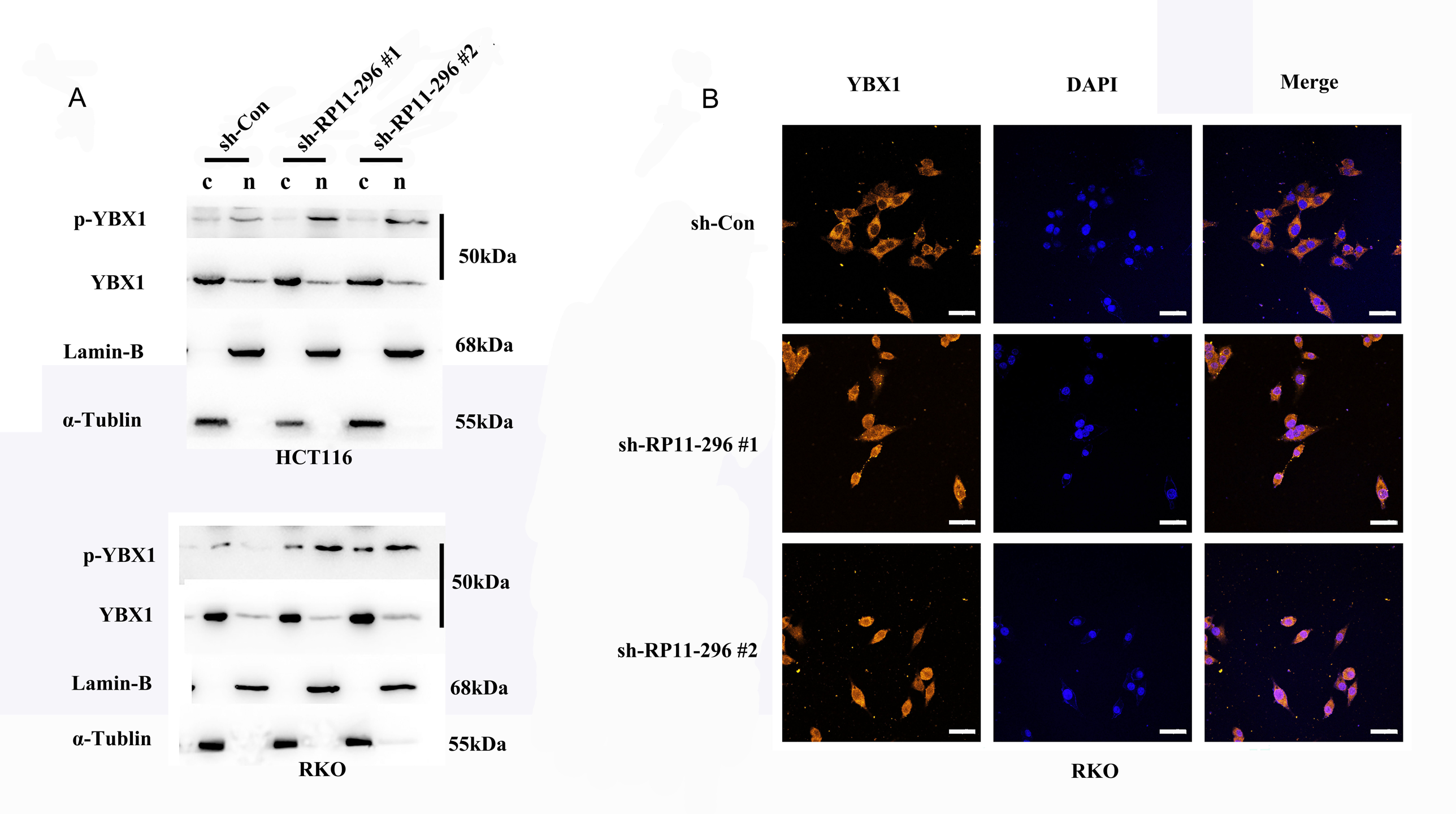

Supplement: Supplementary file 5 — Additional file 5: Figure S5. Knockdown of RP11-296E3.2 increased the level of nuclear p-YBX1. (A) WB analysis showed that knockdown of RP11-296E3.2 induced an increase in nuclear p-YBX1 in HCT116 and RKO cells. (B) Confocal microscopy analysis of YBX1 in RKO cells. Knockdown of RP11-296E3.2 induced nuclear accumulation of p-YBX1 (Scale bars: 40 μm). [file 12967_2023_4267_MOESM5_ESM.jpg]
