# Supplementary material for: RP11-296E3.2 acts as an important molecular chaperone for YBX1 and promotes colorectal cancer proliferation and metastasis by activating STAT3
Source: J Transl Med. 2023 Jun 27;21:418. doi: 10.1186/s12967-023-04267-4 (PMC10303830; doi:10.1186/s12967-023-04267-4)
Supplement: Supplementary file 6 — Additional file 6: Table S1. Primers for qRT-PCR. Table S2. Primers for vectors construction. Table S3. Antibodies. [file 12967_2023_4267_MOESM6_ESM.docx]

**Table S1. Primers for qRT-PCR**

| Primer Names | Sequences (5'-3') |
| --- | --- |
| *RP11-296E3.2* Forward | CCAAGACCCAAGGACAACT |
| *RP11-296E3.2* Reverse | GAAGGATGGGCAGGAGATG |
| *JAK2* Forward | TGGGGTTTTCTGGTGCCTTT |
| *JAK2* Reverse | CGCATAAATTCCGCTGGTGG |
| *STAT3* Forward | TCTCCTCCACCACCAAGCGA |
| *STAT3* Reverse | AAGCCCTTGCCAGCCATGTT |
| *STAT5A* Forward | CAGACCAAGTTTGCAGCCAC |
| *STAT5A* Reverse | CACAGCACTTTGTCAGGCAC |
| *YBX1* Forward | GGGGACAAGAAGGTCATCGC |
| *YBX1* Reverse | CGAAGGTACTTCCTGGGGTTA |
| *ELAVL1* Forward | GGGTGACATCGGGAGAACG |
| *ELAVL1* Reverse | CTGAACAGGCTTCGTAACTCAT |
| *ITGAX* Forward | AGAGCTGTGATAAGCCAGTTCC |
| *ITGAX* Reverse | AATTCCTCGAAAGTGAAGTGTGT |
| *MMP1* Forward | AAAATTACACGCCAGATTTGCC |
| *MMP1* Reverse | GGTGTGACATTACTCCAGAGTTG |
| *HDAC9* Forward | AGTAGAGAGGCATCGCAGAGA |
| *HDAC9* Reverse | GGAGTGTCTTTCGTTGCTGAT |
| *LTBP1* Forward | CTGACGGCCACGAACTTCC |
| *LTBP1* Reverse | GCACTGACATTTGTCCCTTGA |
| *GAPDH* Forward | GCACCGTCAAGGCTGAGAAC |
| *GAPDH* Reverse | TGGTGAAGACGCCAGTGGA |
| *RP11-296E3.2-*GSP-5’-1(RACE) | GTGGATACTCATGGGGCAGTGACCAACC |
| *RP11-296E3.2*-GSP-5’-2(RACE) | ACCAACCTGACTGAAGGGTGGGTGC |
| *RP11-296E3.2*-GSP-3’-1(RACE) | ACTTCCGTCAGCCCCACCTCTTTTCTG |
| *RP11-296E3.2*-GSP-3’-2(RACE) | TCCCATTGATTTCAGACGTGCACCCACC |
| *T7-RP11-296E3.2-*sence-(RNA pulldown)- Forward | AGAACCCACTGCTTACTGGCTTATCG |
| *T7-RP11-296E3.2*-sence-(RNA pulldown)- Reverse | TCTAGACTCGAGCGGCCGCCACTGT |
| *T7-RP11-296E3.2-*antisence-(RNA pulldown)- Forward | CCAAGCTGGCTAGCGTTTAAACTTAAGC |
| *T7-RP11-296E3.2-*antisence-(RNA pulldown)- Reverse | TATATTTAATACGACTCACTATAGGGTAGACTCGAGCGGCCGCCACTGTGC |
| *T7-RP11-296E3.2(1-104)-*(RNA pulldown)- Reverse | AGAACCCACTGCTTACTGGCTTATCG |
| *T7-RP11-296E3.2(1-104)-*(RNA pulldown)- Forward | GAATAATAAGCAGTTAAATAATTA |
| *T7-RP11-296E3.2(1-124)-*(RNA pulldown)- Forward | AGAACCCACTGCTTACTGGCTTATCG |
| *T7-RP11-296E3.2(1-124)-*(RNA pulldown)- Reverse | GTATTTAAAAATTTTTAAATGGGC |
| *T7-RP11-296E3.2(125-399)*-(RNA pulldown)- Forward | TATATTTAATACGACTCACTATAGGGTAAAAATTTTTAAATA |
| *T7-RP11-296E3.2(125-399)*-(RNA pulldown)- Reverse | GTGGGTGCACGTCTGAAATCAATG |
| *T7-RP11-296E3.2(400-672)*-(RNA pulldown)- Forward | CCACTAATACGACTCACTATAGGCCTTCAGTCAGGTTGG |
| *T7-RP11-296E3.2(400-672)*-(RNA pulldown)- Reverse | TCTAGACTCGAGCGGCCGCCACTGT |
| *T7-RP11-296E3.2(549-672)*-(RNA pulldown)- Forward | GACTTAATACGACTCACTATAGGCTAAATGACAGTCTGTTC |
| *T7-RP11-296E3.2(549-672)*-(RNA pulldown)- Reverse | TCTAGACTCGAGCGGCCGCCACTGT |
| *STAT3-*promoter-Forward-1(CHIP) | CGGAAACATGACGTGGAAAT |
| *STAT3-*promoter-Reverse-1 (CHIP) | TACACCGTCTTCTGCATTCG |
| *STAT3-*promoter-Forward-2 (CHIP) | CACATGGTTCCCCAGATACC |
| *STAT3-*promoter-Reverse-2 (CHIP) | TCACCACCAGTGACCCTATG |
| *STAT3-*promoter-Forward-3 (CHIP) | AATTGGGGGCTTGGTAAAAA |
| *STAT3-*promoter-Reverse-3 (CHIP) | GGGATCTCTTGCGACTTAGG |
| *STAT3-*promoter-Forward-4 (CHIP) | TCACTCATCCTCATGCCAAG |
| *STAT3-*promoter-Reverse-4 (CHIP) | CAGCTCAGGACAGCTTTGTG |

**Table S2. Primers for vectors construction**

| Primer Names | Sequences (5'-3') |
| --- | --- |
| pET-23(+)-*RP11-296E3.2*-Forward | CGGCTAGCTTTGGCTTCATCTTTATTCA |
| pET-23(+)-*RP11-296E3.2*- Reverse | CCAAGCTTGCAGGTCCTCATTTCTACGT |
| pET-23(+)-*YBX1*- NotI-Forward | TTGCGGCCGCATGAGCAGCGAGGCCGAGACCCAG |
| pET-23(+)-*YBX1*- XbaI-Reverse | GCTCTAGACTCAGCCCCGCCCTGCTCAGCCTC |
| pLKO.1-*shRP11-296E3.2*#1-  Forward | CCGGagaatgttaccaagacccaagCTCGAGcttgggtcttggtaacattctTTTTTTGA |
| pLKO.1- *shRP11-296E3.2*#1-  Reverse | AATTCAAAAAAagaatgttaccaagacccaagCTCGAGcttgggtcttggtaacattct |
| pLKO.1- *shRP11-296E3.2*#2-  Forward | CCGGgatgatttctgtactttaaatCTCGAGatttaaagtacagaaatcatcTTTTTTGAAT |
| pLKO.1- *shRP11-296E3.2*#2-  Reverse | AATTCAAAAAAgatgatttctgtactttaaatCTCGAGatttaaagtacagaaatcatc |
| PGL3-*STAT3*- Forward | GGGGTACCCCGAGGGAAACAGAG |
| PGL3-*STAT3*- Reverse | GAAGATCTTCGTCGGAACCCCCGG |
| Pxj40-*YBX1*- Forward (IP) | CGGGATCCATCACCGCAACCATGA |
| Pxj40-*YBX1*- Forward (IP) | TTGCGGCCGCGGCATTTACTCAGCC |

**Table S3. Antibodies**

| Antibodies | brand | Cat number |
| --- | --- | --- |
| anti-human-JAK2 | Cell Signaling Technology | 3230 |
| anti-human-Phospho-JAK2  (Tyr1007/1008) | Cell Signaling Technology | 3771 |
| anti-human-STAT3 | Cell Signaling Technology | 9139 |
| anti-human-Phospho-STAT3(Tyr705) | Cell Signaling Technology | 9145 |
| anti-human-STAT5A | Cell Signaling Technology | 4807 |
| anti-human-Phospho-STAT5A(Tyr694) | Cell Signaling Technology | 4322 |
| anti-human-YBX1 (WB, IHC) | Cell Signaling Technology | 4202 |
| anti-human-YBX1 (IP, RIP) | Cell Signaling Technology | 9744 |
| anti-human-YBX1 (CHIP) | ABclonal | A3534 |
| anti-human- Phospho -YBX1(Ser102) | Cell Signaling Technology | 2900 |
| anti-human-ELAVL1 | Cell Signaling Technology | 12582 |
| anti-human-E-cadherin | Santa Cruz Biotechnology | sc-8426 |
| anti-human-AKT | Proteintech | 60203-2-Ig |
| anti-human -Phospho-AKT (Ser473) | Proteintech | 80455-1-RR |
| anti-human -Vimentin | Santa Cruz Biotechnology | sc-6260 |
| anti-human-Slug | Santa Cruz Biotechnology | sc-166476 |
| anti-human-GAPDH | Affinity | AF7021 |
| anti-human-ａ-Tubulin | Proteintech | 80762-1-RR |
| anti-human-Lamin-B | Proteintech | 12987-1-AP |
| Alexa Fluor^TM^ 555 donkey Anti-Rabbit IgG (H+L) | invitrogen | A31572 |
